# Boron Crosslinking Enables Anion‐Lean Solvation and Stable Interfaces for Dendrite‐Free Lithium Metal Batteries

**DOI:** 10.1002/advs.78679

**Published:** 2026-11-09

**Authors:** A Hyeon Cho, Hyeoksu Kwon, Yubhin Cho, Seon Min Park, Junsu Son, Ke Zhang, Tae Kyung Lee, U Hyeok Choi

**Affiliations:** ^1^ Department of Polymer Science and Engineering and Program in Environmental and Polymer Engineering Inha University Incheon Republic of Korea; ^2^ Department of Organic and Nano Engineering Hanyang University Seoul Republic of Korea; ^3^ Human‐Tech Convergence Program and Clean‐Energy Research Institute Hanyang University Seoul Republic of Korea; ^4^ MIIT Key Laboratory of Critical Materials Technology for New Energy Conversion and Storage, School of Chemistry and Chemical Engineering Harbin Institute of Technology Harbin China; ^5^ Resource‐Recycling Electronic Materials Laboratory Inha University Incheon Republic of Korea

**Keywords:** anion‐lean solvation, gel polymer electrolyte, lithium metal battery, single‐ion conduction, sp^2^‐hybridized boron

## Abstract

Achieving high‐performance and safe lithium metal batteries (LMBs) requires electrolytes that simultaneously optimize solvation chemistry, interfacial stability, ionic conductivity, and mechanical robustness. Conventional gel polymer electrolytes (GPEs) often suffer from low lithium‐ion transference numbers and sluggish interfacial kinetics because mobile anions and strong Li^+^ solvation induce concentration polarization and dendritic Li growth. Herein, we design a sp^2^‐hybridized boron‐crosslinked gel polymer electrolyte (GPE_TMAB) that integrates anion‐lean solvation and interfacial engineering within a single platform. GPE_TMAB is constructed from a boron‐containing multi‐arm crosslinker (TMAB), methyl ether acrylate, and isobornyl acrylate, together with a binary ethyl methyl carbonate/fluoroethylene carbonate solvent system that balances strong coordination and facile desolvation, thereby establishing soft, Li^+^‐dominant solvation environments. Molecular dynamics simulations reveal that the boron‐mediated TFSI^−^ trapping weakens Li^+^ solvation and enhanced Li^+^ diffusion. Accordingly, Lewis‐acidic boron centers enable Li^+^ single‐ion conduction and promote formation of a mechanically robust, inorganic‐rich SEI. GPE_TMAB achieves high ionic conductivity, intrinsic non‐flammability, dendrite‐free Li plating/stripping for over 1200 h, and durable LFP∥Li full‐cell cycling with 86% capacity retention after 800 cycles, with improved transport kinetics and stable interfacial chemistry. This work establishes a boron‐enabled solvation and interfacial design strategy for high‐voltage, dendrite‐free, and safe all‐solid‐state LMBs.

## Graphical abstract

The boron‐crosslinked gel polymer electrolyte (GPE_TMAB) enhances Li^+^ transport through Lewis‐acidic boron centers that selectively immobilize TFSI^−^ anions, thereby enabling single‐ion conduction and promoting dendrite‐free lithium deposition. The engineered solvation structure drives the formation of an inorganic‐rich, mechanically robust SEI, resulting in exceptional interfacial stability and outstanding cycling performance, establishing GPE_TMAB as a promising platform for next‐generation safe all‐solid‐state batteries.

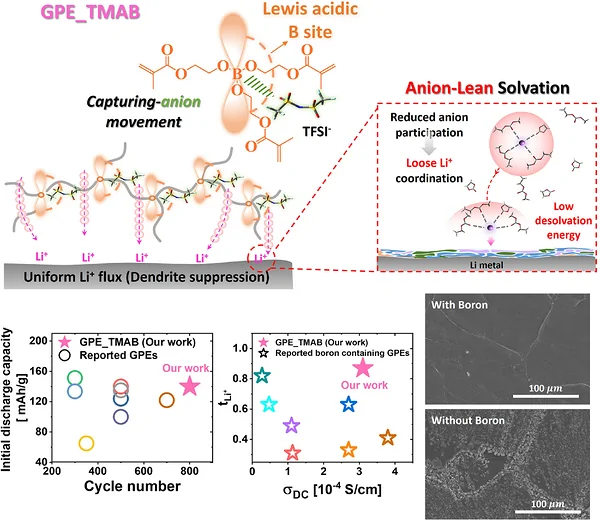

## 1 Introduction

Lithium (Li) metal batteries (LMBs) are regarded as next‐generation energy storage systems due to the ultrahigh theoretical capacity (∼3860 mAh/g) and the lowest redox potential (−3.04 V vs. SHE) of the Li metal anode [1, 2, 3, 4]. However, when coupled with conventional flammable liquid electrolytes (LEs), LMBs suffer from unstable solid electrolyte interphases (SEIs) formation and large volume fluctuations during Li plating/stripping [5, 6, 7]. These factors trigger severe side reactions, uncontrolled dendritic growth, and internal short‐circuiting, leading to critical safety and durability issues that hinder their practical implementation [8, 9, 10].

To overcome these drawbacks, all‐solid‐state LMBs have been developed to simultaneously achieve high energy density and inherent safety. Among various solid‐state electrolytes, solid polymer electrolytes (SPEs) are especially promising due to their flexibility, processability, and ability to maintain intimate interfacial contact with electrodes, compared to inorganic counterparts [11]. However, despite extensive studies, achieving a synergistic combination of high ionic conductivity (essential for fast charging) and mechanical robustness (crucial for suppressing dendrite growth) remains a central challenge. In addition, interfacial instabilities, including the formation of unstable SEIs on Li metal and cathode electrolyte interphases (CEIs) at high voltages, continue to hinder long‐term cycling stability.

Gel polymer electrolytes (GPEs) have emerged as an attractive alternative by combining the high ionic conductivity of LEs with the mechanical integrity of SPEs [12]. They maintain intimate contact with electrodes and effectively buffer electrode volume changes during cycling, thereby mitigating interfacial delamination [13, 14, 15]. However, conventional GPEs still suffer from a low lithium‐ion transference number ($t_{Li^{+}}$) and sluggish interfacial kinetics. The migration of free anions induces concentration polarization and strong local electric fields, which promote parasitic side reactions and Li dendrite formation [16, 17, 18]. Moreover, the strong solvation of Li^+^ by solvent molecules increases the desolvation energy barrier, further hindering interfacial charge transfer and overall ionic transport efficiency [19].

To overcome these issues, recent efforts have focused on solvation‐structure engineering as a powerful route to enhance both ionic transport and interfacial stability in GPEs [20]. Tailoring the solvation shell to modulate the Li^+^‐anion‐solvent coordination environment governs SEI chemistry and Coulombic efficiency (CE). Numerous electrolyte designs based on anion‐enriched solvation structures, obtained from high‐concentration electrolytes (HCEs) and localized high‐concentration electrolytes (LHCEs), have achieved CE values exceeding 99% by forming inorganic‐rich SEIs. However, further improvement is limited because coordinated anions within the solvation shell are preferentially decomposed at the Li metal anode over other components, accelerating anion depletion and Li corrosion (i.e., ramified Li deposition and electrically isolated dead Li), which ultimately deteriorate long‐term performance [21]. Moreover, as recently revealed, anions within the solvation shell donate excess electrons to solvents, lowering their atomic charge and consequently the oxidation potential of the entire electrolyte, which undermines high‐voltage stability [22]. These findings underscore that anion‐decoupled Li^+^ solvation structures are essential for designing high‐voltage, corrosion‐resistant electrolytes while still enabling the formation of robust inorganic‐rich SEIs.

Herein, we propose sp^2^‐hybridized boron‐crosslinked GPEs featuring with Li^+^ single‐ion conduction and anion‐decoupled solvation structure, enabling stable interfacial chemistry and high‐performance operation in LMBs (Figure 1). The electrolyte (referred to as GPE_TMAB) is constructed using a boron‐containing multi‐arm crosslinker, tris(2‐methacryloyloxyethyl) borate (TMAB), copolymerized with methyl ether acrylate (MEA) and isobornyl acrylate (IBA) monomers, and incorporates lithium bis(trifluoromethane)sulfonimide (LiTFSI) salt together with a binary solvent system of fluoroethylene carbonate (FEC) and ethyl methyl carbonate (EMC). First, the Lewis‐acidic boron centers selectively immobilize TFSI^−^ anions and suppress direct Li^+–^TFSI^−^ association, thereby creating an *anion‐lean solvation* environment characterized by reduced anion participation in Li^+^ coordination, which facilitates rapid Li^+^ migration and minimizes interfacial polarization. Second, the incorporation of the IBA monomer, containing bulky bicyclic isobornyl groups, together with the flexible MEA comonomer, produces a polymer matrix that achieves a balanced combination of elastic compliance (to accommodate electrode volume changes) and rigidity (to mechanically suppress dendrite growth). This tough‐yet‐flexible structure, enriched with carbonyl oxygen Li^+^ coordination sites, provides a favorable mechanical‐ionic balance for prolonged cycling stability in LMBs. Third, the optimized EMC/FEC combination, possessing strong and weak solvating abilities, respectively, establishes a soft solvating structure that balances coordination strength and facile desolvation, thereby promoting uniform Li deposition and the formation of an inorganic hetero‐SEI layer with high ionic conductivity and mechanical robustness [23, 24]. As a result, the as‐constructed freestanding GPE_TMAB exhibits high ionic conductivity (3.1 × 10^−4^ S/cm at 30°C), a robust shear storage modulus (1.6 × 10^5^ Pa at 25°C), and a high lithium‐ion transference number ($t_{Li^{+}}$ = 0.87 at 25°C), along with excellent nonflammability. Consequently, the GPE_TMAB‐based Li∥Li symmetric cell demonstrates remarkable cycling stability for over 1200 h with uniform Li plating/stripping behavior. Additionally, an LFP∥Li full cell employing GPE_TMAB delivers highly reversible capacity with 86% retention after 800 cycles at 30°C. Overall, this boron‐enabled solvation and interfacial design present a versatile strategy for developing safe, high‐voltage, and dendrite‐free solid‐state LMBs.

**FIGURE 1:**
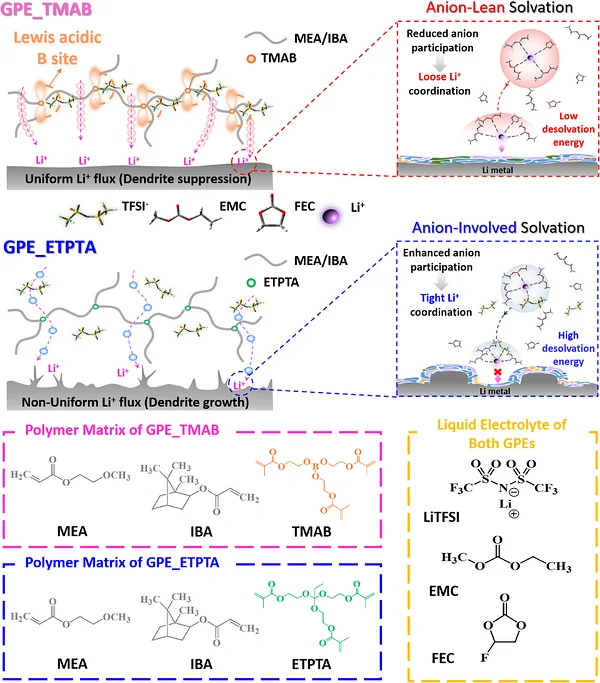
Schematic illustration of the chemical structures, solvation behaviors, and interfacial Li‐ion transport mechanisms in GPE_TMAB and GPE_ETPTA. GPE_TMAB incorporates a Lewis‐acidic boron‐containing crosslinker (TMAB) within a flexible MEA/IBA polymer matrix, forming an anion‐trapping network that immobilizes TFSI^−^ and promotes the formation of a soft, anion‐lean solvation shell. This loose solvation structure lowers the Li^+^ desolvation energy, enables uniform Li^+^ flux, and facilitates the growth of a stable and compact SEI, effectively suppressing dendrite formation. In contrast, GPE_ETPTA, based on an oxygen‐rich ETPTA crosslinked matrix lacking boron sites, allows strong Li^+–^anion coordination, resulting in a tight, anion‐involved solvation shell with higher desolvation energy. The resulting non‐uniform Li^+^ flux and unstable SEI promote dendritic Li growth. The bottom panels present the polymer network compositions of each GPE and the common liquid electrolyte components (FEC/EMC with LiTFSI), highlighting how chemical structure‐driven solvation structure regulation enables interfacial engineering toward high‐performance, dendrite‐free Li metal batteries.

## 2 Results and Discussion

### 2.1 Synthesis of Boron‐Crosslinked Non‐Flammable GPEs

The synthesis, structural confirmation, morphological features, and safety characteristics of the single‐Li^+^ conducting GPE were systematically investigated to evaluate its suitability as a robust electrolyte for LMBs. TMAB was synthesized via a substitution reaction using 2‐hydroxyethyl methacrylate (HEMA) and trimethyl borate (TMB), as described in Figure S1 [25, 26]. The ^1^H NMR analysis was conducted to confirm the successful preparation of TMAB (Figure S2), consistent with previously reported results in the literature [26]. As shown in Figure 1, a single‐Li^+^ conducting GPE (denoted as GPE_TMAB) was prepared via thermal copolymerization of MEA and IBA monomers with a boron‐containing TMAB crosslinker, incorporating weakly coordinating LiTFSI salt and a mixture of EMC and FEC plasticizers with strong and weak solvating abilities, respectively. The combination of flexible MEA units enhances ionic mobility, while bulky IBA units provide mechanical robustness, enabling a balanced GPE matrix with both high conductivity and interfacial stability. For comparison, a dual‐ion conducting GPE (denoted as GPE_ETPTA) was prepared using ethoxylated trimethylolpropane triacrylate (ETPTA) as the crosslinker, which resembles TMAB in chemical structure but lacks the boron moiety (Figure S3), while employing the same monomers, salt, and plasticizers as GPE_TMAB. The detailed compositions of each GPE are summarized in Table S1.

The transparent and flexible GPE_TMAB monolithic film was successfully obtained via a facile radical polymerization, as shown in Figure 2a, demonstrating its ability to be easily folded and rolled under mechanical deformation. To verify polymer network formation by the crosslinker, GPE_TMAB was compared with and without TMAB after polymerization (Figure S4). As shown, the TMAB‐containing solution underwent successful gelation (Figure S4a), whereas the system without TMAB remained liquid (Figure S4b). Fourier transform infrared spectroscopy (FTIR) analysis was also conducted to confirm the crosslinking reaction of GPE_TMAB (Figure 2b). The absorption peak observed at 1637 cm^−1^, assigned to the vibration of the vinyl group (C═C) in IBA, MEA, and TMAB, disappeared after crosslinking, indicating successful completion of the polymerization.

**FIGURE 2:**
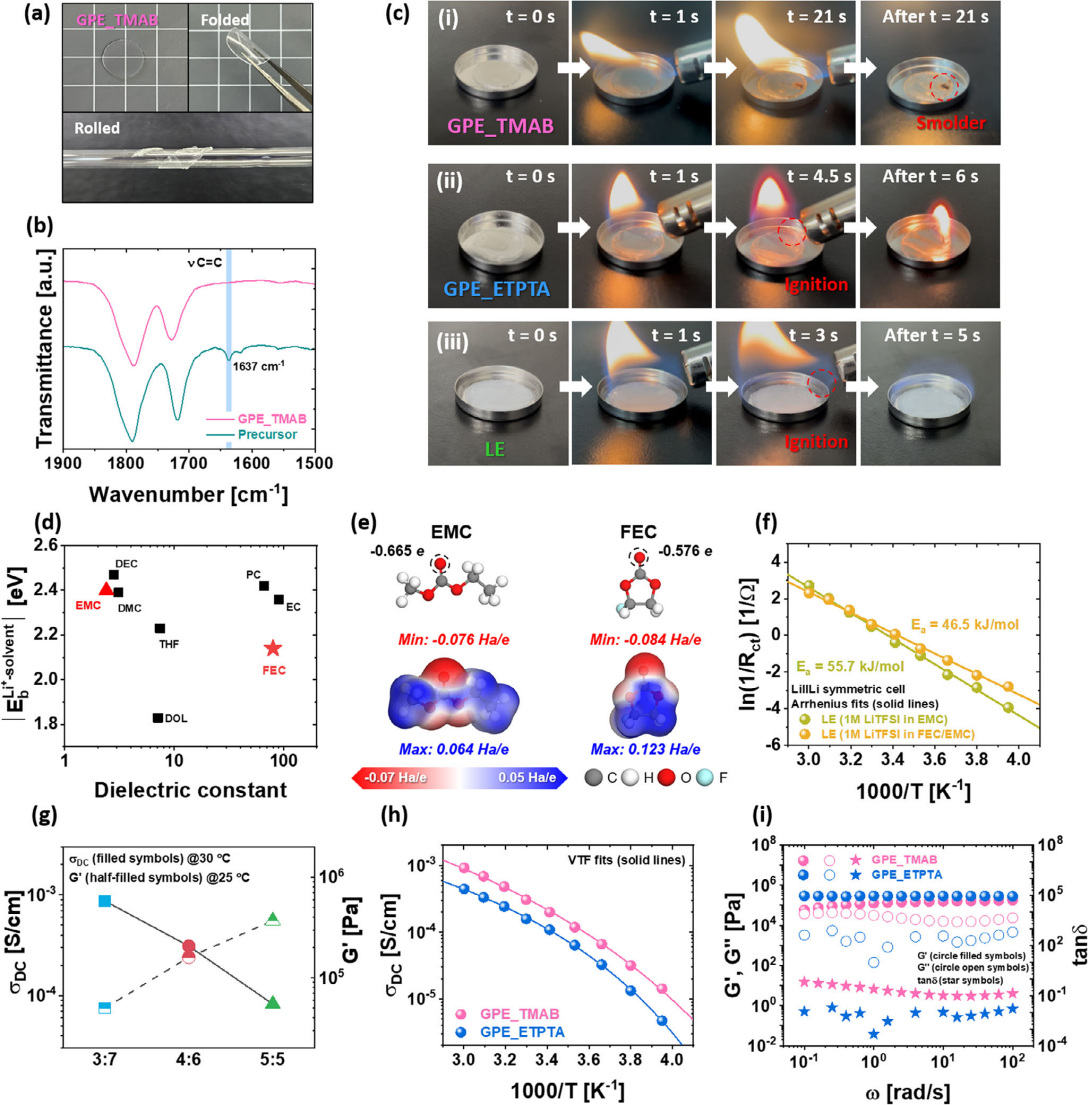
(a) Optical images of flexible and transparent GPE_TMAB film. (b) FTIR spectra of GPE_TMAB and its precursor. (c) Optical images of the flammability tests for GPE_TMAB, GPE_ETPTA, and liquid electrolyte (LE, 1 M LiTFSI‐FEC/EMC with a PP separator). Videos S1‐S3 provide the corresponding flammability test recordings. (d) Li^+^‐solvent binding energy versus dielectric constant for various solvents. (e) Electrostatic potential (ESP) charge and ESP isosurface of EMC (left) and FEC (right), and the isovalue of ESP isosurface is 0.03 *e*/Å^3^. (f) Arrhenius plots of 1/R_ct_ values at different temperatures (from −20°C to 60°C) for Li symmetric cells of LE (1 M LiTFSI–EMC) and LE (1 M LiTFSI–FEC/EMC). The activation energies for interfacial charge transfer processes were obtained from the slope of the fitted line. (g) Comparison of ionic conductivity σ_DC_ (filled symbols, left y‐axis) at 30°C and storage moduli *G*′ (half‐filled symbols, right y‐axis) at room temperature according to the ratio of polymer matrix to liquid electrolyte in GPE_TMAB. (h) Temperature dependence of ionic conductivity σ_DC_ for GPE_TMAB and GPE_ETPTA. Solid lines are VTF fits to data. (i) Angular frequency dependence of storage modulus (*G*′, filled symbols, left y‐axis), loss modulus (*G*′′, open symbols, left y‐axis), and loss factor (tanδ, star symbols, right y‐axis) for GPE_TMAB and GPE_ETPTA at room temperature.

The morphology of GPE_TMAB was investigated by field emission scanning electron microscope (FE‐SEM) (Figure S5). Figure S5a displays a compact cross‐section with a thickness of 180 µm, while the inset of Figure S5b shows a highly ordered wrinkle structure that facilitates intimate contact with Li metal and accommodates volume changes during cycling [27]. To confirm the distribution of TMAB and LiTFSI, the energy dispersive spectroscopy (EDS) analysis was carried out (Figure S6). Figure S6a shows the B element mapping, indicating uniform dispersion of TMAB within GPE_TMAB. Such a homogenous distribution of crosslinking points ensures complete polymer network formation and offers uniform sites for Lewis acid‐base interactions between the sp^2^‐hybridized boron atoms and TFSI^−^ anions. Furthermore, the uniform distribution of other elements (such as C, N, O, F, and S) indicates that all components are well distributed in GPE_TMAB.

The thermal stability of GPE_TMAB was investigated to determine its safe operating temperature range for LMBs. As shown in Figure S7, thermogravimetric analysis (TGA) indicates that the 5% weight‐loss temperature ($T_{d}^{5\%}$, indicated by dashed lines) occurs at approximately 110°C, demonstrating excellent thermal stability. This result suggests that GPE_TMAB can operate reliably at elevated temperatures, supporting its potential for high‐temperature LMB applications. To further ensure safety against fire hazards, the non‐flammability of GPE_TMAB was also evaluated. As shown in Figure 2c–i and Video S1, even after a 20‐second ignition attempt, GPE_TMAB exhibited no ignition, with only minor surface charring, maintaining its original form without deformation. In contrast, GPE_ETPTA, which shares a similar crosslinked structure but lacks boron, ignited within approximately 4.5 s under the same conditions (Figure 2c‐ii and Video S2). This stark difference is attributed to the uniformly distributed boron in GPE_TMAB (Figure S6), which forms boron oxide (B_2_O_3_) during combustion. The resulting glassy protective layer on the polymer surface effectively blocks oxygen ingress and suppresses further combustion [28]. For comparison, the non‐crosslinked liquid electrolyte (LE, composed of EMC/FEC (1:1 vol%) with 1 M LiTFSI) ignited immediately and sustained combustion upon exposure to flame (Figure 2c‐iii and Video S3). Consequently, GPE_TMAB demonstrates superior thermal stability and inherent flame resistance, offering a robust safety advantage by mitigating fire risks. This highlights GPE_TMAB as an effective electrolyte design strategy for enhancing the thermal and fire safety of LMBs.

### 2.2 Design Strategy of GPE: From Solvation Chemistry to Ion Transport

Optimizing the performance of LMBs requires precise control of Li^+^ conductivity and the solvation environment, both within the electrolyte and at the electrolyte/electrode interface. This necessitates careful electrolyte design that accounts for solvent properties to ensure stable operation under fast‐charging and high‐voltage conditions. Key parameters such as Li^+^ binding energy, dielectric constant, donor number (DN), and viscosity are critical for theoretically tailoring electrolyte solvation structures and tuning Li^+^ desolvation kinetics (Table S2) [29, 30, 31]. Guided by this framework, we first calculated the binding energies between Li^+^ and various solvents using density functional theory (DFT) calculations to identify candidates that enable rapid Li^+^ transport.

Figure 2d shows the dielectric constant (ε) and absolute binding energies ($|E_{b}^{{\mathrm{Li}}^{+}-\mathrm{solvent}}|$) of different solvents with Li^+^ ions. Unlike PC and EC, which exhibit both high ε (> 60) and strong $E_{b}^{Li^{+}-\mathrm{solvent}}$ (<−2.30 eV), FEC combines a high ε = 79.7 with a relatively low DN (9.1) and weaker Li^+^ binding energy (−2.14 eV, Figure S8) [32]. These apparently different trends arise because the dielectric constant primarily reflects the bulk electrostatic screening ability of the solvent, whereas Li^+^ binding strength is governed by the local Lewis basicity of the coordinating site. The high dielectric constant of FEC facilitates LiTFSI dissociation by screening Li^+–^TFSI^−^ electrostatic interactions, while the electron‐withdrawing fluorine substituent lowers the electron density of the carbonyl oxygen and weakens its coordination with Li^+^. This interpretation is consistent with previous reports showing that a combination of low DN, weak binding strength, and high dielectric constant induces moderate binding strength together with sufficient salt dissociation [33], and that electrolytes with low dielectric constant exhibit reduced salt dissociation, leading to the formation of contact ion pairs and aggregated ion species [34]. In contrast, EMC possesses a low dielectric constant (ε = 2.4) but a higher DN (17.2) and stronger Li^+^ binding energy (−2.40 eV). Accordingly, the combination of FEC and EMC provides sufficient salt dissociation while avoiding excessively strong Li^+^ solvation, thereby establishing a soft solvation environment favorable for facile Li^+^ desolvation. Furthermore, electrostatic potential (ESP) charge and ESP isosurface reveal that the carbonyl oxygen in EMC carries a more negative charge (−0.665 *e*) than that in FEC (−0.576 *e*), leading to stronger interactions with Li^+^. EMC, with |ESP isosurface_min_| > ESP isosurface_max_, concentrates negative charge on oxygen atoms, enabling strong Li^+^ binding, whereas FEC, with |ESP isosurface_min_| < ESP isosurface_max_, exhibits more delocalized negative charge (Figure 2e).

As a weak electron donor, FEC only partially coordinates with Li^+^, thereby weakening EMC‐Li^+^ interactions and facilitating Li^+^ desolvation, while also offering good SEI‐forming ability and high oxidative stability. Thus, EMC ensures stable Li^+^ coordination, while the electron‐withdrawing fluorine atoms in FEC reduce solvation strength and enable easier desolvation. The complementary roles of EMC (strong coordination) and FEC (facile desolvation with efficient salt dissociation) synergistically balance Li^+^ solvation and desolvation, ultimately promoting rapid Li^+^ transport and high ionic conductivity in GPE_TMAB. Together, the use of strongly solvating EMC and weakly solvating FEC effectively lowers the desolvation energy of our GPE. To validate this hypothesis, the charge transfer resistance (*R*_ct_) of Li symmetric cells based on different liquid electrolytes (with and without FEC) was measured over a temperature range of −20°C to 60°C and analyzed using the Arrhenius equation:

(1) $$R_{\mathrm{ct}}=R_{0}\exp\left[-\frac{E_{a}^{\mathrm{Arr}}}{RT}\right]\;$$

where *R*_0_ is the pre‐exponential factor reflecting the effective interfacial area and charge‐carrier density exponential, $E_{a}^{\mathrm{Arr}}$ represents the desolvation energy barrier, *R* is the ideal gas constant, and *T* is the absolute temperature. As shown in Figure 2f, the desolvation energy, obtained from Arrhenius fits to the *R*_ct_ data (solid lines) decreases from 55.7 kJ/mol for EMC alone to 46.5 kJ/mol when EMC is combined with FEC. This reduction confirms that FEC, acting as a weakly coordinating cosolvent, facilitates Li^+^ desolvation by weakening the strong EMC–Li^+^ interactions. This interpretation is further supported by the molecular dynamics (MD) simulation results discussed later, which reveal that increased FEC participation in the Li^+^ solvation shell weakens Li^+–^solvent interactions and promotes a less tightly bound solvation structure.

To optimize the electrolyte for LMBs, the ionic conductivity (reflecting fast‐charging capability) and viscoelastic properties (reflecting tolerance to volume expansion/contraction) of GPE_TMAB were systematically investigated at different polymer matrix‐to‐liquid electrolyte ratios (Figures S9 and S10). Figure 2g compares the DC ionic conductivity (σ_DC_, filled symbols, measured at 30°C) and shear storage modulus (*G*′, half‐filled symbols, measured at 25°C) of GPE_TMAB with polymer matrix‐to‐liquid electrolyte ratios of 3:7, 4:6, and 5:5. Among them, the 4:6 ratio exhibits the best balance, with σ_DC_ = 3.1 × 10^−4^ S/cm and *G*′ = 1.6 × 10^5^ Pa. In contrast, the 5:5 ratio shows insufficient ionic conductivity (σ_DC_ = 8.2 × 10^−5^ S/cm), while the 3:7 ratio shows poor mechanical strength (*G*′ = 5.0 × 10^4^ Pa). Therefore, the 4:6 composition was selected as the optimal electrolyte formulation for LMB application. Next, beyond the effect of polymer matrix‐to‐liquid electrolyte ratio, the influence of the boron moiety on ionic conductivity and viscoelastic properties was examined by comparing GPE_TMAB (with boron) and GPE_ETPTA (without boron).

Figure 2h shows the temperature dependence of σ_DC_ for GPE_TMAB and GPE_ETPTA over the range of −20°C to 60°C. GPE_TMAB exhibits higher σ_DC_, reaching 3.1 × 10^−4^ S/cm at 30°C and 1.4 × 10^−5^ S/cm even at −20°C, compared to GPE_ETPTA (1.5 × 10^−4^ S/cm at 30°C and 4.7 × 10^−6^ S/cm at −20°C). The temperature dependence of σ_DC_(*T*) for both GPEs follows the Vogel‐Tammann‐Fulcher (VTF) equation:

(2) $$\sigma_{\mathrm{DC}}\left(T\right)=AT^{-\frac{1}{2}}\exp\left[-\frac{E_{a}^{\mathrm{VTF}}}{R\left(T-T_{0}\right)}\right]$$

wherein *A*, $E_{a}^{\mathrm{VTF}}$, *R*, and *T*_0_ (listed in Table S3) are the pre‐exponential factor, the activation energy of ion conduction, the gas constant, and the Vogel temperature, respectively. The good agreement of the data with VTF fits further confirms the polymer‐segmental‐motion‐assisted ion transport mechanism in these GPEs. The activation energy $E_{a}^{\mathrm{VTF}}$ of GPE_TMAB exhibits a lower value of 4.65 kJ/mol, while GPE_ETPTA shows a higher value of 4.97 kJ/mol. This result indicates that the rapid migration of Li^+^, facilitated by a reduced conduction energy barrier, originates from the Lewis acid‐base interaction between GPE_TMAB and TFSI^−^, thereby promoting efficient Li^+^ conduction [35, 36, 37]. Furthermore, as shown in Figure 2i, both GPE_TMAB and GPE_ETPTA exhibit elastic‐dominant viscoelastic behavior [*G*′ (circle filled symbols) > *G*′′ (circle open symbols) and tanδ < 1 (star symbols)], characteristic of well‐formed crosslinked networks. Although GPE_ETPTA shows a slightly higher shear storage modulus (*G*′ = 2.8 × 10^5^ Pa at 25°C) than GPE_TMAB (*G*′ = 1.6 × 10^5^ Pa at 25°C), GPE_TMAB exhibits a higher loss factor (tanδ), suggesting greater chain mobility and viscoelastic relaxation. This enhanced relaxation facilitates Li^+^ hopping through dynamic polymer segments and allows the electrolyte matrix to effectively buffer local volume fluctuations at the Li/electrolyte interface during cycling. Consequently, GPE_TMAB achieves an optimal balance between ionic mobility and mechanical resilience, enabling efficient Li^+^ conduction and suppressing dendrite growth for long‐term stable operation in LMBs.

### 2.3 Unveiling the Anion‐Trapping Role of Boron Moieties Toward Superior Li^+^ Transport

To elucidate the Lewis acid‐base interactions between the boron in TMAB and TFSI^−^ in LiTFSI, DFT calculations were conducted. In Figure 3a, the binding energy (*E*_b_) of Li^+^ with TFSI^−^ is−0.801 eV for LiTFSI, while its complex with the crosslinked TMAB reduces the magnitude of *E*_b_ to −0.691 eV for LiTFSI‐TMAB. The weakened binding energy indicates that the Lewis acid‐base interaction between boron and TFSI^−^ weakens the LiTFSI ion‐pair, thereby promoting Li^+^ dissociation. These results were further confirmed through ^11^B NMR analysis of the corresponding mixture. Figure 3b shows the broad peak at 19.60 ppm, corresponding to the characteristic signal of tri‐oxygen‐coordinated boron (Figure S1) [38]. Upon addition of LiTFSI, the boron chemical shift exhibits a downfield shift, indicating enhanced interactions between TFSI^−^ anions and B atoms in TMAB, which increases the electron density around boron. To further probe these Lewis acid‐base interactions, FTIR spectroscopy was conducted on GPE_TMAB. In Figure S11, LiTFSI exhibits absorption peaks observed at 1205 and 1143 cm^−1^, corresponding to the ‐CF_3_ and ‐SO_2_ stretching vibrations of TFSI^−^, respectively [39, 40]. In GPE_TMAB, these peaks shift to lower wavenumber from 1205 to 1190 cm^−1^ and 1143 to 1136 cm^−1^, consistent with weakened Li^+–^TFSI^−^ binding caused by coordination between TFSI^−^ anions and boron atoms in TMAB‐containing polymer matrix.

**FIGURE 3:**
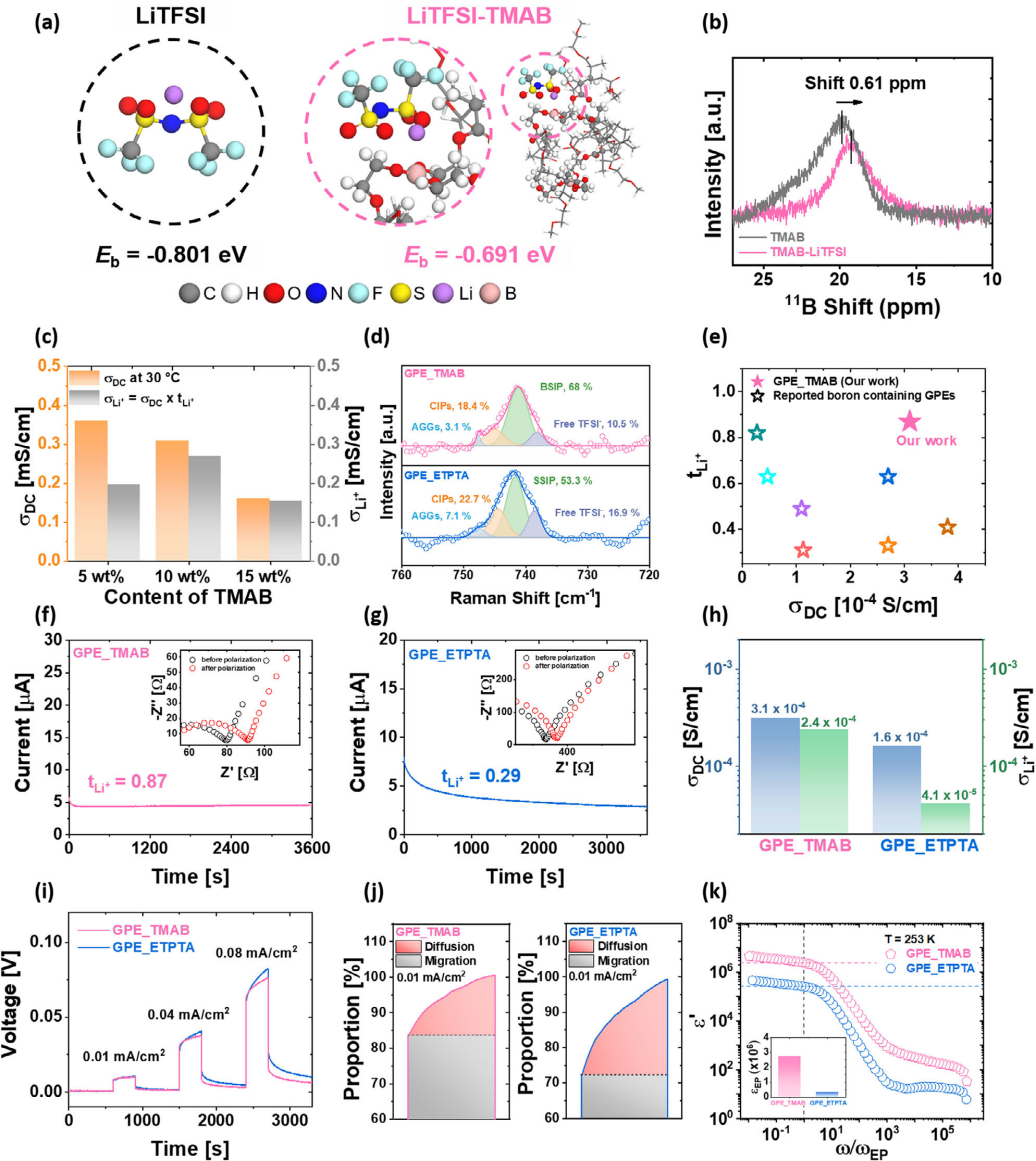
(a) DFT‐calculated molecular structures and Li^+^ binding energies (*E*_b_) of TFSI^−^ anion and crosslinked TMAB‐TFSI^−^ complexes. (b) ^11^B NMR spectra of TMAB and LiTFSI–TMAB mixture. (c) Ionic conductivity (σ_DC_, orange bar, left y‐axis) and Li^+^ conductivity ($\sigma_{\mathrm{DC}}\times t_{Li^{+}}$, gray bar, right y‐axis) comparison of GPE_TMAB with different TMAB contents (5 wt.%, 10 wt.%, and 15 wt.%). (d) Raman spectra of GPE_TMAB and GPE_ETPTA, with deconvoluted peaks assigned to aggregated ion species (AGGs), contact ion pairs (CIPs), free TFSI^−^ anions, and boron‐ or solvent‐separated ion pairs (BSIPs or SSIPs), respectively. (e) Comparison of our GPE_TMAB with reported boron containing GPEs (listed in Table S4) in terms of σ_*DC*_ and $t_{Li^{+}}$ [38, 42, 43, 44, 45, 46, 47]. Li^+^ transference number profiles of (f) GPE_TMAB and (g) GPE_ETPTA: current as a function of time for Li||Li symmetric cells during a 10‐mV polarization experiment at 25°C, with an inset showing the AC impedance spectra of the cell before and after polarization. (h) Ionic conductivity (σ_DC_, blue bar, left y‐axis) and Li^+^ conductivity ($\sigma_{\mathrm{DC}}\times t_{Li^{+}}$, green bar, right y‐axis) comparison of GPE_TMAB and GPE_ETPTA. (i) Voltage profiles of Li∥Li symmetric cells based on GPE_TMAB and GPE_ETPTA at current densities of 0.01, 0.04, and 0.08 mA/cm^2^. (j) Normalized voltage profiles for (left) GPE_TMAB‐ and (right) GPE_ETPTA‐based Li∥Li symmetric cells at a current density of 0.01 mA/cm^2^. (k) Dielectric permittivity ε′(ω/ω_*EP*_) with respect to angular frequency normalized by electrode polarization frequency ω_*EP*_ (indicated by the dashed vertical line) of GPE_TMAB and GPE_ETPTA at 253 K. The dashed horizontal lines indicate ε′(ω_*EP*_/ω_*EP*_)  = ε_*EP*_ , which is the dielectric permittivity after electrode polarization is complete, and the inset compares ε_*EP*_ between GPE_TMAB and GPE_ETPTA.

To verify the anion‐capturing capability of TMAB within the GPE, the Li^+^ transference number ($t_{Li^{+}}$) was measured for GPE_TMAB with varying TMAB contents (5, 10, and 15 wt.%). As shown in Figure S12, $t_{Li^{+}}$ increases from 0.55 to 0.87 and 0.96 with higher TMAB content, approaching almost unity at 15 wt.%. These results clearly demonstrate the strong TFSI^−^ anion‐capturing ability of TMAB. To further assess the effect of high $t_{Li^{+}}$ on ion transport, Figure 3c compares the total ionic conductivity (σ_DC_, orange bar symbols), which accounts for both cations and anions transport, with the single‐Li^+^ conductivity ($\sigma_{Li^{+}}=\sigma_{\mathrm{DC}}\;\times t_{Li^{+}}$, gray bar symbols) for GPE_TMAB with different TMAB loadings. All GPE_TMABs exhibit high σ_DC_, exceeding 10^−4^ S/cm at 30°C. With increasing TMAB (crosslinker) content, σ_DC_ gradually decreases, presumably due to reduced segmental motion from additional crosslinking points, whereas $\sigma_{Li^{+}}$ exhibits a non‐monotonic trend. Notably, the GPE_TMAB containing 10 wt% TMAB achieves the highest $\sigma_{Li^{+}}$, demonstrating superior Li^+^ transport capability.

In addition, Raman spectroscopy was further conducted to explore the anion‐trapping properties of GPE_TMAB. As shown in Figure 3d, the peaks at approximately 738, 741, 745, and 747.9 cm^−1^ are the contributions of free TFSI^−^, boron‐ or solvent‐separated ion pair (BSIPs or SSIPs), contact ion pairs (CIPs), and aggregated ion species (AGGs), respectively [26, 41]. In the case of GPE_TMAB, the dominant BSIP fraction (68%) clearly indicates that the introduction of boron moieties promotes strong Lewis acid‐base interactions with TFSI^−^ anions, thereby effectively weakening Li^+^‐TFSI^−^ coordination. As a result, both the fractions of free TFSI^−^ (10.5%), which can contribute to concentration polarization, and CIPs (18.4%), which cannot participate in ionic conduction, are significantly reduced compared to GPE_ETPTA (16.9% and 22.7%, respectively). These results demonstrate that Li^+^ transport in GPE_TMAB proceeds primarily through boron‐stabilized BSIP structures, enabling more efficient, Li^+^‐centered ion conduction with suppressed anion mobility. Furthermore, the inclusion of FEC, owing to its high dielectric constant (Figure 2d), significantly enhances the dissociation of LiTFSI within GPE_TMAB by stabilizing separated ion species. In the absence of FEC (Figure S13), the proportion of contact ion pairs (CIPs) increases from 18.4% to 21.3%, while the fraction of boron‐separated ion pairs (BSIPs) decreases from 68% to 58.3%, indicating that Li^+–^TFSI^−^ coordination becomes stronger without FEC. This reduced dissociation efficiency also results in a higher amount of free TFSI^−^ (increasing from 10.5% to 15.7%), as the weakly dissociated salt produces uncoordinated anions that can participate effectively in concentration polarization at the electrolyte/electrode interface. Therefore, the presence of FEC promotes effective salt dissociation and balanced Li^+^ solvation structures, enabling selective and rapid Li^+^ conduction. As a result, GPE_TMAB exhibits superior salt dissociation ability compared to previously reported sp^2^‐hybridized boron‐containing polymer electrolytes (Figure 3e and Table S4) [38, 42, 43, 44, 45, 46, 47], which typically suffer from partial anion trapping, lower ionic conductivity, and moderate Li^+^ transference numbers due to incomplete Li^+–^TFSI^−^ dissociation.

The reduced fractions of free TFSI^−^ and CIPs, along with the predominant formation of BSIPs in GPE_TMAB are consistent with its high Li^+^ transference number achieved through TMAB incorporation. As shown in Figure 3f,g, GPE_TMAB exhibits a $t_{Li^{+}}$ of 0.87, which is three times higher than that of boron‐free GPE_ETPTA ($t_{Li^{+}}$ = 0.29). Figure 3h further compares the total ionic conductivity (σ_DC_, blue bar symbols) and single‐Li^+^ conductivity ($\sigma_{Li^{+}}$, green bar symbols) of the two GPEs at 30°C. GPE_TMAB shows higher values for both conductivities (σ_DC_ and $\sigma_{Li^{+}}$); in particular, its $\sigma_{Li^{+}}$ (2.4 × 10^−4^ S/cm) is nearly six times higher than that of GPE_ETPTA (4.1 × 10^−5^ S/cm), underscoring the superior Li^+^ transport capability enabled by the boron‐containing crosslinker. This enhancement originates from Lewis acid‐base interactions between the anion‐accepting boron moieties and TFSI^−^ anions, which immobilize anions and increase $t_{Li^{+}}$ and $\sigma_{Li^{+}}$. The resulting high Li^+^ transport efficiency facilitates uniform and rapid Li^+^ migration, alleviating concentration polarization and regulating interfacial charge‐transfer kinetics. This leads to homogeneous Li deposition, effective dendrite suppression, and excellent long‐term cycling stability in LMBs.

To evaluate the effect of the anion‐accepting GPEs on concentration polarization, galvanostatic DC polarization tests were conducted using Li∥Li symmetric cells. Theoretically, if $t_{Li^{+}}$ = 1 and ion diffusion (driven by concentration polarization) is absent, the voltage response to a direct current should exhibit an ideal square shape, where the entire voltage rise originates solely from ion migration under an electric field [48, 49]. In contrast, conventional dual‐ion conducting electrolytes exhibit a sloping voltage response under direct current, reflecting the contribution of ion diffusion in addition to migration. The voltage profiles of GPE_TMAB and GPE_ETPTA were measured at low current densities of 0.01, 0.04, and 0.08 mA/cm^2^ at 30°C (Figure 3i). The voltage profile of single‐ion conducting GPE_TMAB containing boron moieties has a more ideal square shape compared to the dual‐ion conducting GPE_ETPTA without boron. For a detailed comparison, Figure 3j magnifies the voltage response at 0.01 mA/cm^2^, separating the contributions of ion diffusion and ion migration. The proportion of voltage increase attributed to ion migration is 84% for GPE_TMAB and 72% for GPE_ETPTA, indicating that the anion‐accepting boron moieties effectively suppress concentration polarization. Similar trends are observed in normalized voltage profiles at 0.01, 0.04, and 0.08 mA/cm^2^ (Figure S14). By suppressing concentration polarization through boron‐mediated anion trapping, GPE_TMAB enables rapid and uniform movements of Li^+^, thereby enhancing LMB performance.

To further validate the superior Li^+^ transport of GPE_TMAB, dielectric measurements were conducted to probe electrode polarization (EP) at the electrode/electrolyte interface. This approach reveals how mobile ions accumulate at the electrode/electrolyte interface, with the detailed analysis provided in Supporting Information (Figure S15). Figure 3k shows the dielectric permittivity with respect to frequency normalized by EP frequency ε′(ω/ω_*EP*_) for GPE_TMAB and GPE_ETPTA at 253 K. At ω  = ω_*EP*_ (indicated by the dashed vertical line in Figure 3k), the boron‐containing GPE_TMAB exhibits ε_*EP*_ =  2.7 × 10^6^, approximately 10 times higher than that of GPE_ETPTA (ε_*EP*_ =  3.2 × 10^5^, inset of Figure 3k). This result indicates that Li^+^ ions in GPE_TMAB are more effectively dissociated from TFSI^−^ anions and accumulate more uniformly at blocking electrodes. Such behavior arises from Lewis acid‐base interactions between boron and TFSI^−^, which promote ion‐pair dissociation and enable more Li^+^ ions to migrate toward the electrode, thereby contributing to the superior ionic transport efficiency of GPE_TMAB. Moreover, using a simple resistor‐capacitor circuit model, we quantitatively calculated the surface charge density (Q/A), representing the charge density of migrating ions accumulating on the electrode surface, as given by the following equation:

(3) $$\frac{Q}{A}=\frac{V\sigma_{DC}}{\omega_{EP}L}$$

where *Q* is the charge within the stern layer, *A* is the surface area of the electrode, *V* is the applied voltage, and *L* is the thickness of the electrolyte. Figure S15b shows the charge density at the electrode calculated by Equation (3). GPE_TMAB exhibits a significantly higher charge density value (*Q*/*A*  =  1.37 × 10^−4^ C/m^2^), which is approximately 10 times greater than that of GPE_ETPTA (*Q*/*A*  =  1.34 × 10^−5^ C/m^2^). Therefore, all these theoretical and experimental results demonstrate that Lewis acid‐base interactions between boron and TFSI^−^ effectively suppress TFSI^−^ migration, allowing more Li^+^ ions to reach the electrode uniformly. Furthermore, the efficient ion‐pair dissociation lowers the desolvation energy at the Li metal interface, promoting homogeneous lithium deposition.

### 2.4 MD Simulation Insights Into Boron‐Regulated Solvation Structures and Li^+^ Transport

To gain molecular‐level insight into the effects of TMAB crosslinker incorporation on Li^+^ solvation structures and TFSI^−^ anion trapping, MD simulations and DFT calculations are performed. The GPE_TMAB and GPE_ETPTA systems are constructed through the crosslinking process (Figures S16–S18a and Table S5). Following the application of an external electric field to both systems (Figure S18b), the Li^+^ solvation free‐energy is increased in the GPE_TMAB system than in the GPE_ETPTA system, indicating more facile Li^+^ desolvation in the GPE_TMAB system (Figure 4a). To understand origin of the facile Li^+^ desolvation in the GPE_TMAB system, the radial distribution function (RDF) analysis of Li^+^ coordination with liquid electrolyte components was conducted. In GPE_ETPTA (Figure 4c), the coordination number (CN) between Li^+^ and EMC [Li^+–^O (EMC)] is noticeably higher than that between Li^+^ and FEC [Li^+–^O (FEC)], indicating that the Li^+^ solvation structure is predominantly governed by strongly coordinating EMC molecules. In contrast, GPE_TMAB exhibits comparable CN values for Li^+^–O (EMC) and Li^+^–O (FEC) interactions (Figure 4b), suggesting that weakly coordinating FEC molecules participate more actively in the Li^+^ solvation shell. This balanced coordination environment forms a less tightly bound Li^+^ solvation structure, thereby facilitating Li^+^ desolvation in the GPE_TMAB system. Moreover, in both GPE_TMAB and GPE_ETPTA systems, the magnitude of the Li^+^ binding energies with solvents decreases in the 2EMC‐2FEC solvation structures than in the 3EMC‐1FEC solvation structures, indicating that the increased FEC coordination weakens Li^+^–solvent interactions and thereby facilitates Li^+^ desolvation (Figures S19 and S20).

**FIGURE 4:**
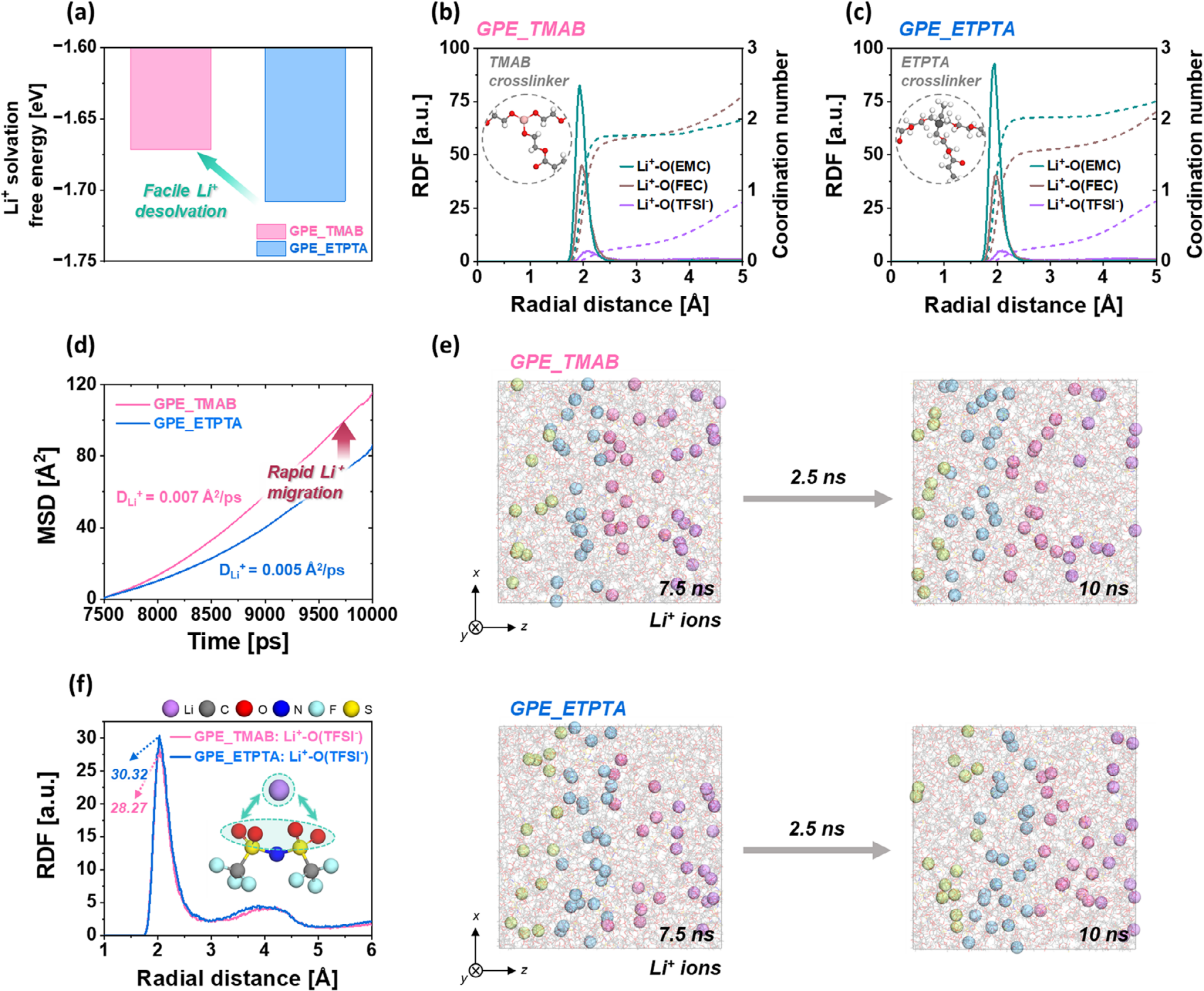
(a) Solvation free energies of Li^+^ ions in the GPE_TMAB and GPE_ETPTA systems. For clarity, the solvation free energies of Li^+^ ions are normalized by the number of Li^+^ ions. (b,c) Solvation structures of Li^+^ ions characterized by radial distribution functions (RDFs, solid lines) and coordination numbers (CNs, dashed line) in (b) the GPE_TMAB and (c) GPE_ETPTA systems obtained from MD simulations. (d) Mean squared displacement (MSD) and diffusion coefficients of Li^+^ ions in GPE_TMAB and GPE_ETPTA systems during the last 2.5 ns of the MD simulations. (e) Trajectories of Li^+^ ions in the GPE_TMAB and GPE_ETPTA systems during the last 2.5 ns of the MD simulations. Note that the Li^+^ ions are distinguished by four different colors to clearly visualize their movement. (f) RDFs of Li^+–^TFSI^−^ in solvent‐free GPE_TMAB and GPE_ETPTA with LiTFSI.

Additionally, Li^+^ diffusion behavior was investigated by the analysis of mean squared displacement (MSD). A steeper MSD slope is observed for the GPE_TMAB system compared with the GPE_ETPTA system, suggesting faster Li^+^ diffusion (Figure 4d,e). Consistently, the Li^+^ diffusion coefficient in the GPE_TMAB system is approximately 1.4 times higher than that in the GPE_ETPTA system, confirming enhanced Li^+^ transport, which can be attributed to TMAB crosslinker incorporation and the resulting TFSI^−^ anion trapping effect. Given that the CNs of TFSI^−^ are similar in the GPE_TMAB and GPE_ETPTA systems, the solvent‐free model systems are further employed to elucidate Li^+–^TFSI^−^ coordination and the effects of TFSI^−^ trapping on Li^+^ diffusion (Figure 4b,c, Figure S21a, and Table S5). The RDF results show that the probability of Li^+–^TFSI^−^ coordination in the GPE_TMAB system is reduced compared to the GPE_ETPTA system (Figure 4f and Figure S21b). In consistent with the MD simulation results, the crosslinked TMAB–TFSI^−^ exhibits weaker binding strength with Li^+^ compared to the crosslinked ETPTA–TFSI^−^ (Figure S22). TFSI^−^ anion shows the stronger binding affinity toward crosslinked TMAB than crosslinked ETPTA (Figure S23). It can be attributed to the boron sites of TMAB crosslinker, which possess more positive ESP charges than the central carbon sites of the ETPTA crosslinker, enabling stronger electrostatic attraction toward TFSI^−^ anion (Figure S24). These findings confirm that the boron sites of the TMAB crosslinker effectively facilitate TFSI^−^ anion trapping, which promotes Li^+–^TFSI^−^ dissociation and consequently enhances the Li^+^ migration. Overall, our results highlight that boron‐containing TMAB crosslinker effectively modulates Li^+^ solvation structures and enhances Li^+^ transport through strengthened TFSI^−^ trapping within the gel polymer electrolyte.

### 2.5 Solvation‐Driven SEI Chemistry and Dendrite Suppression

For long‐life LMBs, it is essential to design an electrolyte that is electrochemically stable and capable of suppressing Li dendrite growth on the lithium metal. To investigate the electrochemical stability of GPE_TMAB, linear sweep voltammetry (LSV) tests were carried out using Li∥stainless steel (SS) cells. Figure 5a shows LSV curves of GPE_TMAB and GPE_ETPTA with a scan rate of 1 mV/s at 30°C. GPE_TMAB exhibits a high oxidation voltage up to 5.1 V versus Li^+^/Li, exceeding that of GPE_ETPTA (4.8 V), indicating its significantly wider electrochemical stability window (ESW). This broad stability window further suggests that GPE_TMAB can support the stable operation of high‐voltage LMBs. To elucidate the wide ESW of GPE_TMAB, DFT calculations were performed to investigate the effect of boron on the molecular energy levels. Figure 5b displays the highest occupied molecular orbital (HOMO) energy levels of crosslinked TMAB, crosslinked ETPTA, LiTFSI, EMC, and FEC. The boron‐containing crosslinked TMAB exhibits a lower HOMO level compared to the boron‐free crosslinked ETPTA. It indicates that the TMAB can effectively improve the electrochemical stability compared to ETPTA, consistent with the LSV results.

**FIGURE 5:**
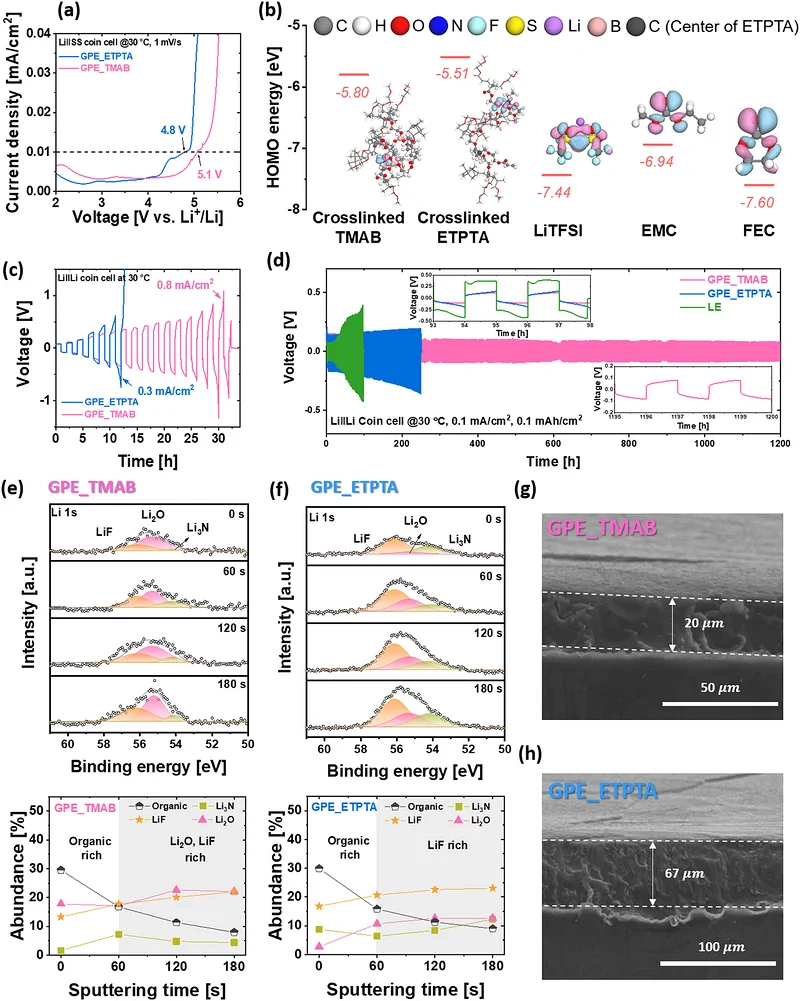
(a) LSV curves of Li∥SS coin cells assembled with GPE_TMAB and GPE_ETPTA within a scan rate of 1 mV/s at 30°C. (b) HOMO energy level diagram of crosslinked TMAB, crosslinked ETPTA, LiTFSI, EMC, and FEC. (c) Voltage profiles indicating the CCD of Li∥Li symmetric coin cells assembled with GPE_TMAB and GPE_ETPTA within a current density increasing by 0.05 mA/cm^2^ at 30°C. (d) Voltage profiles of the Li||Li symmetric cells with GPE_TMAB, GPE_ETPTA, and LE at a current density of 0.1 mA/cm^2^ with a stripping/plating period of 1 h at 30°C, with insets showing magnified views of each profile. Li 1s XPS depth profiles of (e) GPE_TMAB and (f) GPE_ETPTA and their absolute intensities of organic, Li_3_N, Li_2_O, and LiF within the formed SEI layer. Cross‐sectional SEM images of Li metal after 50 cycles with (g) GPE_TMAB and (h) GPE_ETPTA at 0.1 mA/cm^2^ and 0.1 mAh/cm^2^.

To evaluate the ability of GPE to suppress lithium dendrite formation, galvanostatic Li stripping/plating measurements were conducted using Li∥Li symmetric cells at 30°C. The current density started at 0.05 mA/cm^2^ and was increased in 0.05 mA/cm^2^ increments, with each step maintained for 1 h. As shown in Figure 5c, GPE_TMAB maintains stable voltage profiles up to 0.8 mA/cm^2^, whereas GPE_ETPTA shows a short circuit at a lower current density of 0.3 mA/cm^2^. The higher critical current density (CCD) of GPE_TMAB suggests its ability to suppress TFSI^−^ anions migration, thereby enabling rapid and uniform Li^+^ plating/stripping on the Li metal surface. In contrast, GPE_ETPTA, with its low $t_{Li^{+}}$, is more susceptible to concentration polarization, leading to non‐uniform Li^+^ flux. To further assess Li dendrite suppression during long‐term cycling and the reversible transfer of Li^+^ across the electrolyte and electrode interfaces, Li plating/stripping tests were performed at 0.1 mA/cm^2^ with a deposition capacity of 0.1 mAh/cm^2^ at 30°C (Figure 5d). The GPE_TMAB‐based Li∥Li cell shows a stable Li stripping/plating curve for 1200 h, whereas irreversible voltage drops occur in GPE_ETPTA‐ and LE‐based cells after 250 and 100 h, respectively. Moreover, GPE_TMAB maintains stable cycling for over 900 h at an elevated current density of 0.2 mA/cm^2^ and capacity of 0.2 mAh/cm^2^ (Figure S25). These results demonstrate that GPE_TMAB effectively suppresses Li dendrite growth on the surface of Li metal, delivering far superior cycle stability compared to LE. In particular, the single‐ion conducting GPE_TMAB, with suppressed concentration polarization, achieves remarkable long‐term stability in Li∥Li symmetric cells, due to the fast and uniform migration of Li^+^.

X‐ray photoelectron spectroscopy (XPS) depth‐profiling analysis was performed to examine the interfacial chemical composition of the SEI formed on the Li metal, collected from a Li symmetric cell assembled with GPE at 0.1 mA/cm^2^ and 0.1 mAh/cm^2^ after 50 cycles. As shown in Figure S26a, the F 1s XPS spectrum of GPE_TMAB exhibits only a LiF peak at 685.2 eV, whereas GPE_ETPTA (Figure S27a) also displays an additional CF_3_ peak at 687 eV. This distinct difference is further confirmed in the C 1s spectrum, where GPE_ETPTA displays a clear CF_3_ signal (Figure S27c) that is absent in GPE_TMAB (Figure S26c). The exclusive detection of CF_3_ organic byproduct in GPE_ETPTA indicates that LiF originates from the reductive decomposition of TFSI^−^ within its tight solvation shell (Figure 1). In contrast, the absence of CF_3_ in GPE_TMAB suggests that its LiF species mainly arises from FEC reduction within a loose solvation shell (Figure 1), since most TFSI^−^ is immobilized by coordination with boron and excluded from solvation. Accordingly, the LiF in the SEI layer primarily derives from FEC reduction in GPE_TMAB and from TFSI^−^ reduction in GPE_ETPTA. These different sources of LiF also influence the distribution of other inorganic SEI components. Li 1s XPS depth‐profiling of GPE_TMAB (Figure 5e) and GPE_ETPTA (Figure 5f) reveals that both electrolytes form SEIs containing LiF (56.1 eV), Li_2_O (55.3 eV), and Li_3_N (54.2 eV), but with distinct compositional distributions. In GPE_TMAB, the inner SEI is enriched with heterogeneous inorganic species, with LiF and Li_2_O present in comparable amounts, each exceeding ∼20% abundance. By contrast, the SEI formed in GPE_ETPTA is strongly LiF‐rich, while Li_2_O is limited to a minor fraction (∼10%). LiF is a desirable SEI component for suppressing Li dendrite growth due to its high electrical insulation and mechanical robustness; however, its intrinsically low Li^+^ conductivity limits homogeneous ion transport across the SEI. In contrast, GPE_TMAB generates a hetero‐SEI composed of a balanced mixture of LiF and Li_2_O, where LiF provides mechanical stability for dendrite suppression and Li_2_O contributes higher ionic conductivity. The synergistic effect of this LiF/Li_2_O heterostructure enables both effective dendrite suppression and uniform Li^+^ conduction, leading to a more stable SEI than LiF alone.

Figure 5g,h shows cross‐sectional SEM images of cycled Li metal in Li∥Li cells with GPE_TMAB and GPE_ETPTA after 50 cycles, respectively. In the cell using GPE_TMAB, a uniform and compact Li deposition layer with a thickness of ∼20 µm was observed, indicating stable Li plating/stripping and minimal interfacial fluctuation. In contrast, the cell with GPE_ETPTA exhibited a much thicker (∼67 µm) and unevenly deposited Li layer accompanied by clear signs of interfacial decomposition. This pronounced morphological difference suggests that GPE_TMAB effectively suppresses dendritic growth and side reactions, whereas GPE_ETPTA suffers from unstable interfacial chemistry and uncontrolled Li deposition. Therefore, the distinct solvation structures (loose, FEC‐based in GPE_TMAB versus tight, TFSI^−^‐based in GPE_ETPTA) led to markedly different SEI compositions (LiF/Li_2_O balanced vs. LiF‐dominant), which directly governed Li plating/stripping behavior. As a result, the hetero‐SEI formed in GPE_TMAB effectively suppressed dendrite growth and enabled stable, long‐term cycling, in sharp contrast to the unstable SEI of GPE_ETPTA.

### 2.6 Solvation‐Engineered SEI Enabling Uniform Li Deposition and High Reversibility

To further investigate the lithium deposition behavior under various current density conditions, SEM images were analyzed from Li symmetric cells employing GPE_TMAB and GPE_ETPTA. Figure 6a–f shows the morphology of the lithium metal surface after 50 cycles of Li plating/stripping at current densities of 0.1 and 0.3 mA/cm^2^, with a fixed areal capacity of 0.1 mAh/cm^2^. At a low current density (0.1 mA/cm^2^), relatively uniform lithium surfaces were observed for both GPE_TMAB (Figure 6a) and GPE_ETPTA (Figure 6d), similar to the morphology observed in bare Li (Figure S28). However, at a higher current density (0.3 mA/cm^2^), a clear difference in deposition morphology between the two electrolytes was evident. In the case of GPE_TMAB (Figure 6b), the Li surface remains compact and continuous, with large grains (∼1 mm) and only minor surface texture. The high magnification image (Figure 6c) shows a flat surface with no cracks or mossy/dendritic Li, even at higher current density. In contrast, for GPE_ETPTA (Figure 6e), the Li surface appears rough and discontinuous with smaller grains (∼300 µm) under the same conditions, and the high‐magnification SEM image (Figure 6f) reveals porous deposits with evident dead lithium and dendritic growth along the grain boundaries, indicative of unstable Li plating/stripping.

**FIGURE 6:**
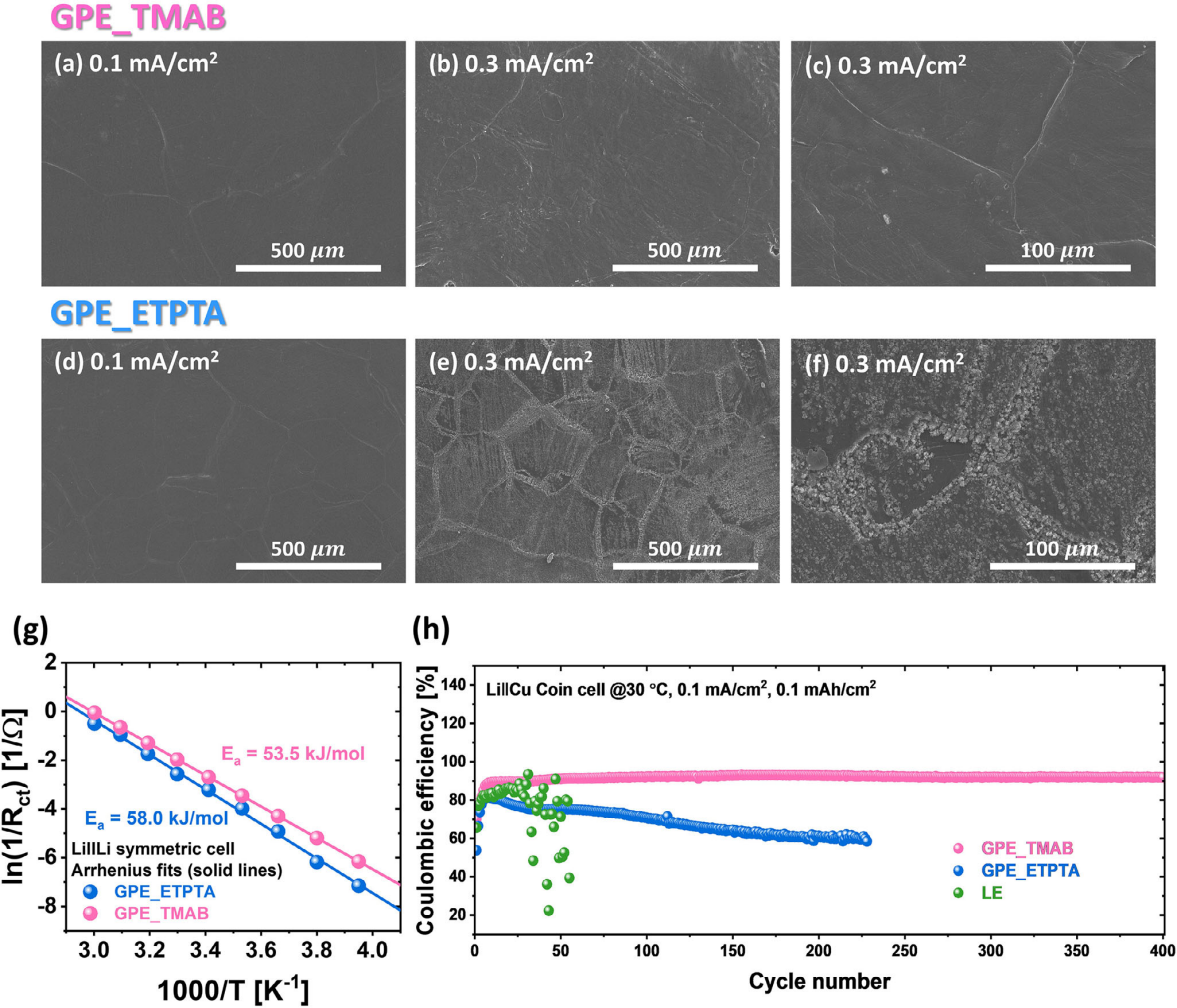
(a–f) SEM images of 50‐cycled Li metal surface with GPE_TMAB (top images) and GPE_ETPTA (bottom images) with 0.1 mA/cm^2^ and 0.3 mA/cm^2^ at 0.1 mAh/cm^2^. (g) Arrhenius plots of 1/R_ct_ values at different temperatures (from −20°C to 60°C) for Li symmetric cells of GPE_TMAB and GPE_ETPTA. The activation energies for interfacial charge transfer processes were obtained from the slope of the Arrhenius‐fitted line. (h) Coulombic efficiencies of Li∥Cu coin cells assembled with three electrolytes (GPE_TMAB, GPE_ETPTA, and LE) within a current density of 0.1 mA/cm^2^ and a deposition capacity of 0.1 mAh/cm^2^ at 30°C.

This morphological difference can be explained by the $t_{Li^{+}}$, which governs lithium deposition characteristics. Generally, when Li^+^ transport is dominated by diffusion, concentration polarization occurs, leading to Li^+^ depletion near the electrode interface, reduced exchange current density, and the need for higher overpotential, ultimately producing smaller grains. Conversely, when lithium‐ion migration dominates, as in single‐ion conducting systems, concentration polarization is suppressed, allowing uniform current distribution at lower overpotentials and enabling the formation of larger grains. In GPE_TMAB, a high $t_{Li^{+}}$ of 0.87 is achieved by suppressing anion mobility through Lewis acid‐base interactions between the boron moiety and TFSI^−^ anions, allowing efficient Li^+^ conduction only. Together with the formation of a balanced LiF/Li_2_O hetero‐SEI (Figure 5e), this promotes uniform Li^+^ flux, reduces dendrite nucleation, and yields stable and compact Li deposits with large grains. Meanwhile, GPE_ETPTA, with a relatively low $t_{Li^{+}}$ of 0.29, permits free anion motion and forms a diffusion‐dominated conduction environment (Figure 3j). Combined with its LiF‐rich SEI (Figure 5f), formed mainly by TFSI^−^ reduction and exhibiting poor ionic conductivity, this leads to enhanced concentration polarization, higher overpotentials, smaller grain sizes, and the growth of dendritic Li metal under higher current density. Indeed, the Tafel plot in Figure S29 shows that the exchange current density of GPE_ETPTA (0.09 mA/cm^2^) is lower than that of GPE_TMAB (0.12 mA/cm^2^), which well supports the diffusion‐dominated environment described above.

To further evaluate interfacial Li^+^ transport, desolvation energy was evaluated using Li symmetric cells assembled with GPE_TMAB and GPE_ETPTA (Figure 6g). Their *R_ct_* values were measured over a temperature range of −20 to 60°C, and activation energies were extracted by Arrhenius fitting (Equation 1). The desolvation energy of GPE_ETPTA is 58.0 kJ/mol, whereas GPE_TMAB shows a lower value of 53.5 kJ/mol, consistent with the MD simulation results that reveal a weaker Li^+^ solvation environment and more facile Li^+^ desolvation in GPE_TMAB (Figure 4a). The lower energy barrier in GPE_TMAB arises from its anion‐lean, loose solvation shell, which accelerates Li^+^ reduction at the electrode and promotes uniform deposition. In contrast, the tight solvation shell in GPE_ETPTA, dominated by strong anion coordination, raises the energy barrier, retarding Li^+^ reduction and leading to non‐uniform Li growth. To further verify the reversibility of Li stripping/plating, the Coulombic efficiency for galvanostatic Li stripping/plating in Li∥Cu cells was next recorded. Figure 6h indicates the Coulombic efficiency of Li∥Cu cells assembled with GPE_TMAB, GPE_ETPTA, or liquid electrolyte (LE), evaluated at a current density of 0.1 mA/cm^2^, a deposition capacity of 0.1 mAh/cm^2^, and 30°C, as a function of cycle number. In the LE‐based Li∥Cu cell, Coulombic efficiency initially reaches ∼80% due to low interfacial resistance but drops sharply after 30 cycles, indicating poor reversibility of Li stripping/plating and insufficient ability to suppress Li dendrite growth. The GPE_ETPTA‐based cell shows more stable cycling for ∼220 cycles without a drastic decline, yet its average Coulombic efficiency remains low (∼69%) due to sluggish and non‐uniform Li^+^ transport. However, the GPE_TMAB‐based cell maintains a high and stable Coulombic efficiency (∼92%) for ∼400 cycles, demonstrating excellent reversibility of Li stripping/plating enabled by fast and uniform Li^+^ migration. These results highlight that tailoring solvation structure and SEI composition, achieved in GPE_TMAB through boron‐mediated anion trapping, provides a powerful design strategy for enabling dendrite‐free Li deposition and highly reversible Li cycling.

### 2.7 From Full Cells to Pouch Cells: Practical Validation of Boron‐Enabled GPEs

To determine the suitability of GPE_TMAB for application to practical LMBs, the electrochemical performance of GPE_TMAB‐based LFP∥Li full cells was evaluated. The galvanostatic charge/discharge (GCD) measurements were first performed to discover the capacity of LFP∥Li cells with a voltage range of 2.5–4.2 V at 30°C. The C‐rate capability of GPE_TMAB‐ and GPE_ETPTA‐based LFP∥Li coin cells was investigated in the range of 0.1–2 C, and the results are shown in Figure 7a. The GPE_TMAB‐based cell delivers discharge capacities of 163.5 mAh/g at 0.1 C and 112.5 mAh/g at 2 C, and notably, after cycling at 2 C, the capacity recovers to 161.8 mAh/g at 0.1 C, corresponding to a recovery rate of ∼99%. In contrast, the GPE_ETPTA‐based cell exhibits lower discharge capacities of 145.2 mAh/g at 0.1 C and 86.7 mAh/g at 2 C, and further suffers from a continuous decline in capacity, with poor recovery upon returning to 0.1 C (retention of only 95%). The GCD curves illustrating the C‐rate performance of GPE_TMAB‐ and GPE_ETPTA‐based LFP∥Li cells are presented in Figure S30. Long‐term cycling stability was carried out by conducting GCD tests using LFP∥Li coin cells assembled with GPE_TMAB and GPE_ETPTA operated at 1 C with cut‐off voltages of 2.5–4.2 V at 30°C (Figure 7b). The discharge capacity of GPE_TMAB‐based LFP∥Li cell is maintained without a significant decrease from the initial discharge capacity over 800 cycles, delivering a high‐capacity retention of 86% and a high average Coulombic efficiency of 99.9%. In contrast, the GPE_ETPTA‐based cell shows unstable capacity retention starting from ∼100 cycles, continuously decreasing to ∼50% at 300 cycles, indicating poor cycling stability. These differences are attributed to the boron‐enabled anion‐trapping and balanced LiF/Li_2_O hetero‐SEI of GPE_TMAB, which synergistically ensures uniform and rapid Li^+^ transport, stable interface, and dendrite‐free cycling, thereby delivering outstanding cycling performance in LMBs.

**FIGURE 7:**
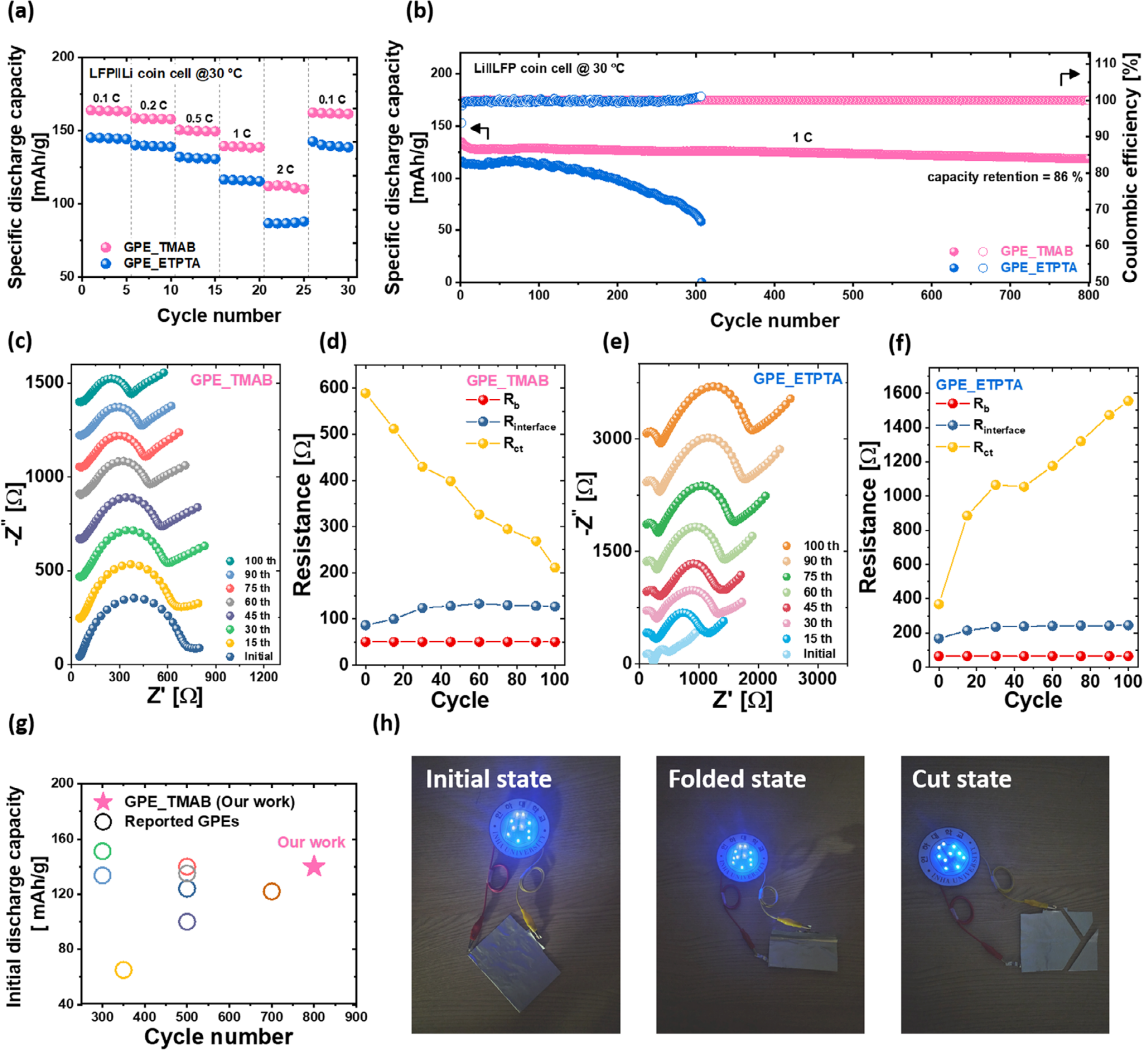
(a) C‐rate capability of GPE_TMAB and GPE_ETPTA in LFP∥Li coin cells at 30°C. (b) Cycle performance of GPE_TMAB and GPE_ETPTA in LFP∥Li coin cells at 30°C. Nyquist plots of the Li|LFP full cells with (c) GPE_TMAB and (e) GPE_ETPTA recorded at various cycles. The corresponding EIS fitting values are shown in (d) and (f), where bulk resistance (*R_b_*), interface resistance (*R_interface_*), and charge transfer resistance (*R_ct_*), were extracted using an equivalent circuit model (Figure S32). (g) Comparison of initial capacity and cycle number at 1 C for our full cell with recently reported GPE results (listed in Table S6) [50, 51, 52, 53, 54, 55, 56, 57]. (h) Optical images of GPE_TMAB‐based LFP∥Li pouch cells illuminating blue LEDs under (left) initial, (middle) 90° folded, and (right) triple‐cut conditions.

Furthermore, the disparity in cycle stability between GPE_TMAB and GPE_ETPTA is evident from the overall cell cyclic voltammetry (CV) tests presented in Figure S31. For the GPE_TMAB‐based LFP∥Li full cell, both cathodic and anodic peaks remain nearly unchanged over five consecutive cycles, indicating excellent reversibility and interfacial stability. In contrast, the GPE_ETPTA‐based cell exhibits noticeable peak shifts and intensity loss, reflecting increased polarization and poor reversibility. These results demonstrate that the boron‐mediated single‐ion conducting GPE_TMAB maintains stable redox kinetics during cycling, whereas GPE_ETPTA suffers from unstable Li^+^ transport and interfacial degradation. To monitor the evolution of resistance during cycling, in‐situ EIS measurements were also performed on LFP∥Li cells using GPE_TMAB (Figure 7c,d) and GPE_ETPTA (Figure 7e,f). From their Nyquist curves, the total resistance of GPE_TMAB gradually decreases over cycling (Figure 7c), whereas that of GPE_ETPTA continuously increases (Figure 7e). To quantitatively identify the origins of these changes, the EIS spectra were fitted using an equivalent circuit model (Figure S32), allowing the resistance to be separated into bulk resistance (*R_b_*), interface resistance (*R_interface_*), and charge transfer resistance (*R_ct_*) components (Figure 7d,f). For both electrolytes, *R_b_* remains stable throughout the cycling, presumably attributed to their wide electrochemical stability (> 4.8 V, Figure 5a) and the electron‐blocking protection provided by the LiF‐rich inner SEI. During the initial cycles, *R_interface_* increases due to SEI formation but stabilizes after approximately 30 cycles. At this stage, the *R_interface_* for GPE_TMAB (∼128 Ω) is significantly lower than that for GPE_ETPTA (∼240 Ω). This can be attributed to the formation of a thinner, more uniform SEI enriched with Li^+^ conducting Li_2_O in GPE_TMAB (Figure 5e), whereas GPE_ETPTA forms a less conductive, LiF‐dominated SEI (Figure 5f). In addition, the *R_ct_* exhibits opposite trends: GPE_TMAB shows a gradual decrease over 100 cycles (Figure 7d), indicating improved charge transfer, whereas GPE_ETPTA exhibits a steady increase (Figure 7f). This contrast is attributed to their different desolvation energies. The lower desolvation energy of GPE_TMAB allows stable Li^+^ availability at the electrode surface, promoting efficient electron transfer and thus decreasing *R_ct_*. In contrast, the higher desolvation energy of GPE_ETPTA causes uneven Li^+^ supply, sluggish interfacial kinetics, and a progressive increase in *R_ct_* during cycling. Figure 7g (summarized in Table S6) compares the initial discharge capacity and cycle life of our GPE_TMAB‐based LFP∥Li full cell with those of recently reported GPEs [50, 51, 52, 53, 54, 55, 56, 57]. The GPE_TMAB‐based cell delivers both a higher initial discharge capacity and much longer cycle life than the state‐of‐the‐art GPEs, which typically show either high capacity with short cycle life or extended cycling with significantly lower capacity. This exceptional combination of high capacity and long‐term durability highlights the effectiveness of our boron‐enabled solvation and interfacial engineering strategy for advancing practical solid‐state LMBs.

To further evaluate the stability of GPE_TMAB with high‐energy‐density electrodes, long‐term cycling tests were conducted using an NCM811|GPE_TMAB|Li full cell. The cell operates stably at 1 C within a voltage window of 3–4.3 V, delivering an initial capacity of 139 mAh/g and retaining 87% of its capacity after 110 cycles (Figure S33). These results confirm that GPE_TMAB enables stable operation even under high‐voltage conditions. Moreover, as demonstrated in Figure 7h, an LFP∥Li pouch cell assembled with GPE_TMAB can successfully light up multiple blue LED lights, which remain illuminated even under mechanical deformation, including folding and cutting of the pouch. These results highlight the robustness and practical applicability of GPE_TMAB for advanced LMBs.

## 3 Conclusions

In this study, we developed a boron‐crosslinked gel polymer electrolyte (GPE_TMAB) featuring Li^+^ single‐ion conduction and anion‐lean solvation chemistry for high‐performance and safe lithium metal batteries (LMBs). The GPE_TMAB, synthesized via copolymerization of the boron‐containing crosslinker TMAB with MEA and IBA monomers, LiTFSI salt, and a binary EMC/FEC solvent system, simultaneously achieves high ionic conductivity (3.1 × 10^−4^ S/cm at 30°C), mechanical robustness (*G*′ = 1.6 × 10^5^ Pa at 25°C), and a high lithium‐ion transference number ($t_{Li^{+}}$ = 0.87). The Lewis‐acidic boron centers effectively immobilize TFSI^−^ anions and promote Li^+^‐dominated ion transport, thereby suppressing concentration polarization and enabling uniform Li plating/stripping. Furthermore, the incorporation of the bulky IBA monomer provides a tough yet flexible polymer network that accommodates interfacial volume fluctuations while maintaining mechanical stability, and the FEC/EMC binary system establishes a soft solvating environment that facilitates facile Li^+^ desolvation and the formation of an inorganic LiF/Li_2_O‐rich SEI. MD simulations further reveal that the boron‐crosslinked structure promotes weakly coordinated Li^+^ solvation and enhanced Li^+^ diffusion through effective TFSI^−^ anion trapping. As a result, GPE_TMAB exhibits outstanding electrochemical and safety characteristics, including high thermal stability ($T_{d}^{5\%}$ = 110°C), intrinsic non‐flammability, and superior cycling durability. Li||Li symmetric cells demonstrate stable cycling for over 1200 h with dendrite‐free morphology, and Li //Cu half‐cells deliver high Coulombic efficiency (∼92%) over 400 cycles, confirming reversible Li plating/stripping. Moreover, LFP||Li full cells exhibit long‐term stability with 86% capacity retention after 800 cycles, and the electrolyte remains mechanically and electrochemically stable even under deformation in pouch‐cell demonstrations. Despite these promising results, future studies under practically relevant conditions, particularly at higher Li plating capacities and cathode loadings, are needed to further establish the practical viability of the boron‐crosslinked GPE platform. Overall, this work establishes a boron‐enabled solvation and interfacial design strategy that unites single‐ion conduction, soft solvation dynamics, and mechanical resilience, offering a promising route toward dendrite‐free, high‐voltage, and safe all‐solid‐state LMBs.

## 4 Experimental section

### 4.1 Materials

2‐Hydroxyethyl methacrylate (HEMA), isobornyl acrylate (IBA), methyl ether acrylate (MEA), lithium bis(trifluoromethane)sulfonimide (LiTFSI), ethoxylated trimethylolpropane triacrylate (ETPTA, average M_n_ ∼ 428 g/mol), dimethyl ether (99%) (DME), and anhydrous acetonitrile were purchased from Sigma Aldrich. Trimethyl borate (TMB), 4‐fluoro‐1,3‐dioxolan‐2‐one (FEC), and ethyl methyl carbonate (EMC) were purchased from Tokyo Chemical Industry. 2,2‐azobisisobutyronitrile (AIBN) was purchased from Junsei Chemical. LiFePO_4_ (LFP) powder, LiNi_0.8_Mn_0.1_Co_0.1_O_2_ (NCM811) powder, conductive acetylene black, N‐methyl‐2‐pyrrolidone (NMP), HSV900 poly(vinylidene fluoride) (PVDF), carbon‐coated Al current collector, and copper foil were purchased from MTI. Lithium metal foil (with a thickness of 200 µm) was purchased from Honjo Metal. The battery separator (Celgard 2320) was purchased from Celgard.

### 4.2 Synthesis of TMAB

For the preparation of TMAB, HEMA (3.9 g, 30 mmol) and TMB (1.0 g, 10 mmol) were dissolved in 50 mL of anhydrous acetonitrile. The mixture was stirred for 4 h at 60°C in a dry N_2_ atmosphere. To eliminate methanol generated as a byproduct, the mixture was stirred for another 2 h at 70°C under N_2_ purging. After stirring, acetonitrile and unreacted materials were removed at 50°C under reduced pressure. The resulting precipitate was vacuum‐dried at room temperature for 2 days, yielding 3.1 g of TMAB with a 78% yield. The detailed preparations of TMAB were described in an earlier study [25, 26] and summarized in Figure S1. The synthesized TMAB was stored in an Ar‐filled glove box to prevent the possibility of hydrolysis. To confirm the TMAB structure, ^1^H NMR analysis was conducted (Figure S2); ^1^H NMR of TMAB: 6.14 (b), 5.60 (b), 4.30‐4.24 (c), 4.02, 3.87 (d), and 1.96 (a).

### 4.3 GPE Preparation

For GPE_TMAB preparation, IBA monomer, MEA monomer, and TMAB crosslinker were added in a liquid electrolyte solution of 1 M LiTFSI in EMC/FEC (1:1 vol%) at a mass ratio of 1:2:1. The mixture was stirred for 1 h at room temperature. Then, an AIBN initiator (1.5 wt.% of monomers and crosslinker) was completely dissolved in the mixture to produce the precursor solution. The electrolyte precursor was polymerized for 2 h at 70°C to obtain a GPE_TMAB film with a reproducible thickness of 180 ± 9 µm (∼5% variation), and films within this thickness range were consistently used for all electrochemical measurements. For GPE_ETPTA, the same procedure was followed, except that the ETPTA crosslinker was used in place of TMAB.

### 4.4 Electrode Preparation and Battery Assembling

To fabricate the LFP cathode, a slurry mixture was prepared by mixing LFP powder as the active material, PVDF binder, and conductive acetylene black in an NMP solvent, following an 8:1:1 mass ratio. The homogeneous slurry prepared by stirring the slurry mixture at room temperature was scraped onto the carbon‐coated Al current collector and dried in a vacuum oven for 2 h at 80°C. Then, a rolling process was performed on the coated slurry to achieve uniform thickness, and the rolled slurry was dried in a vacuum oven at 80°C for 1 day to remove residual NMP solvent. The NCM cathode was fabricated through the same procedure by replacing LFP powder with NCM powder. Finally, the LFP and NCM cathodes were obtained with a mass loading of around 2.0 mg/cm^2^. For electrochemical measurements, coin‐type cells (CR2032) were assembled with as‐prepared LFP and NCM cathodes, modified electrolyte, and Li metal anodes in an Ar‐filled glove box (H_2_O and O_2_ < 0.1 ppm).

### 4.5 Materials Characterization

The ^1^H NMR and ^11^B NMR spectroscopy (Bruker Avance III 400 MHz) analysis was conducted to determine the reaction products using deuterated chloroform (CDCl_3_) solvent at 400 MHz. Fourier transform infrared (FTIR, Shimadzu IRAffinity‐1S) spectra were obtained using an attenuated total reflectance (ATR, PIKE Technologies MIRacle Single Reflection) cell in the spectral range from 600 to 4000 cm^−1^. The morphologies and microstructures were measured by a field emission scanning electron microscope (FE‐SEM, Hitachi S‐4300SE) equipped with an energy dispersive X‐ray (EDX) for elemental mapping. Thermal stability was explored by thermogravimetric analysis (TGA, TA Instruments Q50 thermogravimetric analyzer) with a heating scan rate of 10°C/min from 25°C to 800°C in N_2_ atmosphere. Linear viscoelastic properties of GPE were determined using a rheometer (TA Instruments Discovery HR‐2). Dynamic rheological measurements were performed using the parallel plate geometry with an 8 mm diameter at room temperature. A frequency sweep was carried out within the linear viscoelastic region at a strain of 0.1%, over an angular frequency ranging from 0.1 to 100 rad/s. The Raman spectra were obtained using Horiba LabRAm HR Evolution with 532 nm lasers at room temperature.

### 4.6 Computational Details

#### 4.6.1 Molecular Dynamics (MD) Simulations

To investigate the effects of incorporation of TMAB crosslinker containing boron sites on Li^+^ solvation characteristics and TFSI^−^ anion trapping, MD simulations were performed using COMPASS III forcefield [58]. The electrostatic potential (ESP) charges obtained from the density functional theory (DFT) calculations were assigned as the atomic charges for the electrolyte molecules (*i.e*., Li^+^, TFSI^−^, EMC, and FEC) [59]. Based on previous studies [60, 61], we scaled the atomic charges of Li^+^ and TFSI^−^ using the solvent refractive indices of EMC (1.378) and FEC (1.4). The scaling factor was 0.72 for MD simulations. The van der Waals interactions were evaluated using atom‐based summation method with a cutoff distance of 12.5 Å, while electrostatic interactions were computed using particle‐particle particle‐mesh method with an accuracy of 0.001 kcal/mol. The isothermal‐isobaric ensemble (*NPT* ensemble) was performed using Berendsen thermostat and barostat [62] with a time step of 1 fs, while the canonical ensemble (*NVT* ensemble) was conducted using Berendsen thermostat [62] with a time step of 1 fs. Further details regarding the MD simulations are provided in Note S1 of the Supporting Information.

#### 4.6.2 Density Functional Theory (DFT) Calculations

All DFT calculations were performed using DMol^3^ program [63, 64]. The crosslinked polymer structures were extracted from the MD simulations after completion of the crosslinking procedure. The crosslinked TMAB and ETPTA consist of 2 TMAB, 7 MEA, and 3 IBA units and 2 ETPTA, 8 MEA, and 2 IBA units, respectively. All electron relativistic core treatment and double numerical plus polarization basis set (version 4.4) were adopted for the core electron and atomic orbital basis sets, respectively. Spin‐polarized calculations were performed using Tkatchenko‐Scheffler method for dispersion correction [65]. The convergence criteria for geometry optimization were 1.0 × 10^−5^ Ha for energy, 0.002 Ha/Å for force, and 0.005 Å for displacement. The self‐consistent field was set to 1.0 × 10^−6^ Ha. Further details regarding the DFT calculations are provided in Note S2 of the Supporting Information.

### 4.7 Electrochemical Measurements and Battery Testing

The electrochemical measurements were carried out using electrochemical impedance spectroscopy (EIS, Bio‐Logic Science Instruments VSP‐300). The EIS of electrolytes was measured using either brass||brass, Li||Li, or LFP||Li electrode configurations over a frequency range from 7 MHz to 0.1 Hz. Ionic conductivities σ of electrolytes at various temperature ranges of −20°C–60°C were determined by the following equation: [47, 66]

(4) $$\sigma =\frac{L}{RS}\;$$

wherein *L*, *R*, and *S* are the thickness of the electrolytes, resistance, and surface area of the smaller electrode, respectively. The EIS measurements under different temperatures were performed using a temperature controller (Bio‐Logic Science Instruments ITS).

The Li^+^ transference number ($t_{Li^{+}}$) of electrolytes was determined by the combination of AC impedance and chronoamperometry of polarization (Δ*V* = 10 mV) measurements using Li∥Li symmetric cells at 25°C and calculated by the following equation:

(5) $$t_{Li^{+}}=\frac{I_{s}\left(\Delta V-I_{0}R_{0}\right)}{I_{0}\left(\Delta V-I_{s}R_{s}\right)}$$

wherein *I*_0_, *I_s_*, *R*_0_, and *R_s_* are the initial‐state current, steady‐state current, initial‐state interfacial resistance, and steady‐state interfacial resistance between the Li foil and electrolyte, respectively.

Linear sweep voltammetry (LSV) tests were performed using Li∥stainless steel (SS) cells with Li metal as a counter and reference electrode and SS as a working electrode. The measurements were conducted over a voltage range of 0 to 6 V at a scan rate of 1 mV/s and a temperature of 30°C. The critical current density (CCD) was measured using Li∥Li symmetric cells with GPE_TMAB and GPE_ETPTA. The measurement was conducted by increasing the current density from 0.05 mA/cm^2^ in increments of 0.05 mA/cm^2^, with each step maintained for 1 h.

X‐ray photoelectron spectroscopy (XPS) depth‐profiling analysis was conducted using Nexsa‐G2 XPS. The lithium metal used for XPS depth‐profiling analysis was collected from the Li||Li coin cells GPE_TMAB and GPE_ETPTA after 50 cycles at 0.1 mA/cm^2^ and 0.1 mAh/cm^2^. The collected lithium metal was washed with DME to remove any electrolyte residues and then vacuum‐dried for 10 min. This washing and drying process was repeated three times, followed by an additional 24‐hour vacuum drying step.

The electrochemical measurements were conducted using CR2032‐type coin cells [67]. All assembly of cells was performed in an Ar‐filled glove box (H_2_O < 0.1 ppm). The galvanostatic Li stripping/plating tests of Li∥Li and Li∥Cu cells assembled with electrolytes studied were carried out at 30°C. The galvanostatic charge/discharge (GCD) measurements of LFP∥Li coin‐type cells assembled with electrolytes were conducted using a battery test system (WonATech WBCS3000Ls) and a potentiostat (Bio‐Logic Science Instruments VSP‐300) with cut‐off voltages of 2.5–4.2 V at 30°C.

## Author Contributions

**A Hyeon Cho**: methodology, software, data curation, investigation, validation, formal analysis, writing – original draft, writing – review and editing. **Hyeoksu Kwon**: conceptualization, data curation, investigation, validation, formal analysis, writing – review and editing. **Yubhin Cho**: methodology, software, data curation, formal analysis, writing – original draft, writing – review and editing, visualization. **Seon Min Park**: data curation, formal analysis, investigation, writing – review and editing. **Junsu Son**: software, investigation, writing – review and editing, data curation. **Ke Zhang**: investigation, validation, formal analysis, writing – review and editing. **Tae Kyung Lee**: methodology, software, formal analysis, supervision, funding acquisition, project administration, resources, writing – original draft, writing – review and editing. **U Hyeok Choi**: conceptuallization, methodology, formal analysis, supervision, funding acquisition, project administration, resources, writing – original draft, writing – review and editing.

## Conflicts of Interest

The authors declare no conflicts of interest.

## Supporting information




**Supporting File 1**: advs78679‐sup‐0001‐SuppMat.docx.

**Supporting File 2**: advs78679‐sup‐0002‐VideoS1.mp4.

**Supporting File 3**: advs78679‐sup‐0003‐VideoS2.mp4.

**Supporting File 4**: advs78679‐sup‐0004‐VideoS3.mp4.

## Data Availability

The data that support the findings of this study are available from the corresponding author upon reasonable request.
