# Supplementary material for: Boron Crosslinking Enables Anion‐Lean Solvation and Stable Interfaces for Dendrite‐Free Lithium Metal Batteries
Source: Adv Sci (Weinh). 2026 Nov 9:e78679. Online ahead of print. doi: 10.1002/advs.78679 (PMC13748894; doi:10.1002/advs.78679)
Supplement: Supplementary file 1 [file ADVS-9999-e78679-s001.docx]

Supporting Information

**Boron Crosslinking Enables Anion-Lean Solvation and Stable Interfaces for Dendrite-Free Lithium Metal Batteries**

A Hyeon Cho^a†^, Hyeoksu Kwon^a†^, Yubhin Cho^b,c†^, Seon Min Park^a^, Junsu Son^b,c^, Ke Zhang^d^, Tae Kyung Lee^b,c^* and U Hyeok Choi^a,e^*

^a^ Department of Polymer Science and Engineering and Program in Environmental and Polymer Engineering, Inha University, Incheon 22212, Republic of Korea

^b^ Department of Organic and Nano Engineering, Hanyang University, Seoul 04763, Republic of Korea

^c^ Human-Tech Convergence Program and Clean-Energy Research Institute, Hanyang University, Seoul 04763, Republic of Korea

^d^ MIIT Key Laboratory of Critical Materials Technology for New Energy Conversion and Storage, School of Chemistry and Chemical Engineering, Harbin Institute of Technology, Harbin 150001, China

^e^ Resource-Recycling Electronic Materials Laboratory, Inha University, Incheon 22212, Republic of Korea

^†^ A.H.C., H.K., and Y.C. contributed equally to this work.

* Corresponding Author E-mail: [tklee@hanyang.ac.kr](mailto:tklee@hanyang.ac.kr), [uhyeok@inha.ac.kr](mailto:uhyeok@inha.ac.kr)

**Figure S1**. Synthesis route of TMAB crosslinker.

**Figure S2**. ^1^H-NMR spectrum (400 MHz, CDCl_3_) of TMAB.

**Figure S3**. Chemical structure of ethoxylated trimethylolpropane triacrylate (ETPTA).

**Figure S4**. Optical photographs showing the gelation properties of GPE_TMAB after 2 hours of thermal polymerization at 70 °C: (a) with TMAB crosslinker and (b) without TMAB crosslinker.

**Figure S5**. (a) Cross-section and (b) surface FE-SEM images of GPE_TMAB.

**Figure S6**. Energy-disperse X-ray spectroscopy (EDS) mapping of (a) boron (B), (b) carbon (C), (c) nitrogen (N), (d) oxygen (O), (e) fluorine (F), and (f) sulfur (S) within GPE_TMAB.

**Figure S7**. Thermogravimetric analysis (TGA) curve of GPE_TMAB.

**Figure S8**. DFT-calculated molecular structures, binding energies ($E_{b}$), and interatomic distances of (a) Li^+^–EMC and (b) Li^+^–FEC complexes, with geometries optimized by Gaussian 03 software package using B3LYP functional and 6–311G(d,p) basis set [C (dark gray), H (light gray), O (red), F (light blue), and Li (pink)].

**Figure S9**. Temperature dependence of DC ionic conductivity ($\sigma_{\mathrm{DC}}$) of GPE_TMAB with various matrix-to-LE ratios (3:7, 4:6, and 5:5) was measured from 60 to -20 °C, with solid lines representing fits of the VTF equation.

**Figure S10**. Angular frequency dependence of storage modulus ($G^{'}$, filled symbols) for GPE_TMAB with various matrix-to-LE ratios (3:7, 4:6, and 5:5) at room temperature.

**Figure S11**. FTIR spectra of LiTFSI and GPE_TMAB.

**Figure S12**. Li^+^ transference number profiles of GPE_TMAB with (a) 5 wt% TMAB crosslinker, (b) 10 wt% TMAB crosslinker, and (c) 15 wt% TMAB crosslinker, showing current as a function of time for Li||Li symmetric cells during a 10 mV polarization experiment at 25 °C, along with insets displaying the AC impedance spectra before and after polarization. (d) Angular frequency dependence of storage modulus ($G^{'}$, filled symbols) and loss modulus ($G^{''}$, open symbols) for GPE_TMAB with 5, 10, and 15 wt% TMAB crosslinker at room temperature.

**Figure S13**. Raman spectra of GPE_TMAB without FEC.

**Figure S14.** Voltage profiles of Li$\parallel$Li symmetric cells assembled with (a–c) GPE_TMAB and (d–f) GPE_ETPTA at current densities of 0.01, 0.04, and 0.08 mA/cm^2^, respectively.

**Figure S15.** (a) Dielectric permittivity spectra $\varepsilon'$ (open symbols) of GPE_TMAB and GPE_ETPTA at *T* = 253 K. Solid lines are the Macdonald model (equation S1). (b) Comparison of surface charge density Q/A between GPE_TMAB and GPE_ETPTA.

Dielectric measurements were performed as a function of angular frequency ($\omega$) using stainless steel symmetric cells to analyze the electrode polarization (EP) behavior. At low frequencies, EP occurs as transporting ions have sufficient time to accumulate and polarize at blocking electrodes. The dielectric constant of EP can be defined as $\varepsilon_{EP}=\varepsilon'(\omega_{EP})$, determined from the angular frequency dependence of dielectric permittivity $\varepsilon'(\omega)$ spectra once EP is complete ($\omega=\omega_{EP}$). The EP frequency ($\omega_{EP}$) was obtained by fitting the data with a modified Macdonald model (Figure S14):

$\varepsilon_{EP}^{*}\left( \omega\right)= \frac{\Delta\varepsilon_{EP}}{{(i\omega/\omega_{EP})}^{1-n}+i\omega/\omega_{EP}}$	(S1)

wherein $\Delta\varepsilon_{EP}$ is the dielectric increment of EP, $\omega_{EP}$ is the frequency of EP, and $n$ is the connection with the electrode roughness.

**Figure S16.** Initial model configurations of polymer TMAB and ETPTA matrix systems prior to crosslinking process using molecular dynamics (MD) simulations.

**Figure S17.** Crosslinking process of polymer TMAB and ETPTA matrix systems obtained from MD simulations.

**Figure S18.** (a) Model systems constructed for MD simulations to investigate the solvation characteristics and diffusion behavior of Li^+^ ions in the GPE_TMAB and GPE_ETPTA systems. (b) MD snapshots of the GPE_TMAB and GPE_ETPTA systems under an applied electric field.

**Figure S19.** Average Li^+^ binding energies in the 3EMC-1FEC and 2EMC-2FEC solvation structures obtained from the GPE_TMAB (left) and GPE_ETPTA (right) systems under an applied electric field.

**Figure S20.** Solvation structures of the (a) GPE_TMAB and (b) GPE_ETPTA systems for the calculation of Li^+^ binding energies.

**Figure S21.** (a) Model systems constructed for MD simulations to provide detailed insight into Li^+^–TFSI^-^ coordination in solvent-free GPE_TMAB and GPE_ETPTA with LiTFSI systems. (b) MD snapshots of solvent-free GPE_TMAB and GPE_ETPTA with LiTFSI systems under an applied electric field.

**Figure S22.** Li^+^ binding energies of TFSI^-^ anion, crosslinked TMAB–TFSI^-^, and crosslinked ETPTA–TFSI^-^.

**Figure S23.** Binding energies of TFSI^-^ anion with crosslinked TMAB and ETPTA.

**Figure S24.** Electrostatic potential (ESP) charge and ESP isosurface of crosslinked TMAB and ETPTA. The isovalue of ESP isosurface is 0.03 *e*/Å^3^.

**Figure S25.** Cycling performance of a Li∥Li symmetric cell with GPE_TMAB at 0.2 mA/cm^2^ and 0.2 mAh/cm^2^.

**Figure S26**. XPS depth profiles of (a) F 1s, (b) Li 1s, (c) C 1s, and (d) O 1s within the SEI layer formed on the Li metal from the Li||Li symmetric coin cell with GPE_TMAB after 50 cycles at 0.1 mA/cm^2^ and 0.1 mAh/cm^2^.

**Figure S27**. XPS depth profiles of (a) F 1s, (b) Li 1s, (c) C 1s, and (d) O 1s within the SEI layer on the Li metal from the Li||Li symmetric coin cell with GPE_ETPTA after 50 cycles at 0.1 mA/cm^2^ and 0.1 mAh/cm^2^.

**Figure S28.** SEM image showing the surface morphology of bare Li metal.

**Figure S29.** Tafel plots of Li symmetric cells with GPE_TMAB and GPE_ETPTA.

**Figure S30**. Charge and discharge curves of LFP$\parallel$Li full cells with (a) GPE_TMAB and (b) GPE_ETPTA at different C-rates.

**Figure S31**. The first five CV curves of the LFP||Li cells based on (a) GPE_TMAB and (b) GPE_ETPTA under a rate of 0.1 mV/s from 2.5 to 4.2 V.

**Figure S32**. Equivalent circuit model used for EIS fitting of the Nyquist plots shown in Figures 7c and 7e. The three fitted resistance parameters (R_b_, R_interface_, and R_ct_) are plotted as a function of cycle number in Figures 7d and 7f.

**Figure S33.** Cycling performance of NCM811||Li full cell based on GPE_TMAB operated at 1 C within a voltage range of 3.0 to 4.3 V at 30 °C.

**Table S1.** Detailed Composition of Each Component within the GPE_TMAB and GPE_ETPTA.

| Material | GPE_TMAB | GPE_ETPTA |
| --- | --- | --- |
| IBA [w/w] | 0.10 | 0.10 |
| MEA [w/w] | 0.20 | 0.20 |
| TMAB [w/w] | 0.10 | - |
| ETPTA [w/w] | - | 0.10 |
| LiTFSI [w/w] | 0.11 | 0.11 |
| FEC [w/w] | 0.29 | 0.29 |
| EMC [w/w] | 0.20 | 0.20 |

**Table S2.** Li^+^-Solvent Binding Energy, Dielectric Constant, Donor Number, and Viscosity for Various Solvents. ^[1–4]^

| Solvent | $\mathbf{E}_{\mathbf{b}}^{\mathbf{Li}^{\mathbf{+}}\mathbf{-solvent}}$  [eV] | Dielectric constant [$\boldsymbol{\varepsilon}$] | Donor number | Viscosity at 25 °C [cP] |
| --- | --- | --- | --- | --- |
| FEC | -2.14 | 79.7 | 9.1 | 4.1 |
| EMC | -2.40 | 2.4 | 17.2 | 0.65 |
| DMC | -2.39 | 3.1 | 17.2 | 0.59 |
| EC | -2.36 | 89.8 | 16.4 | 1.93 |
| DEC | -2.47 | 2.8 | 16 | 0.75 |
| PC | -2.42 | 66.1 | 15 | 2.53 |
| DOL | -1.83 | 7.1 | 18 | 0.59 |
| THF | -2.23 | 7.4 | 20 | 0.46 |

We obtained the Li^+^–solvent binding energy using DFT methods with the Gaussian 03 software package using B3LYP functional and 6–311G(d,p) basis set. The binding energy of the specimens was calculated using the following equation: $E_{b}^{\mathrm{Li}^{+}-solvent}=E_{b}^{\mathrm{complex}}-(E_{b}^{\mathrm{Li}^{+}}+E_{b}^{\mathrm{solvent}})$. The dielectric constant, donor number, and viscosity were referenced from the literature. ^[1–4]^

**Table S3.** Fitting Parameters for the Temperature Dependence of Ionic Conductivity Using VTF (eq. 2).

| Sample | A  [S/cm K^1/2^] | $E_{a}^{\mathrm{VTF}}$  [kJ/mol] | T_0_  [K] |
| --- | --- | --- | --- |
| GPE_TMAB | 0.62 | 4.65 | 184 |
| GPE_ETPTA | 0.44 | 4.97 | 185 |

**Table S4.** Comparison of GPE_TMAB with the Reported Boron Containing Polymer Electrolytes in Terms of $\sigma_{\mathrm{DC}}$ and $t_{{Li}^{+}}$^[5–11]^

| No. | Name | $\sigma_{\mathrm{DC}}$ [S cm^-1^] | $t_{{Li}^{+}}$ |
| --- | --- | --- | --- |
| 1 | GPE_TMAB | 3.1 ${\times10}^{-4}$ | 0.87 |
| 2 | PEO-MBO | 2.7 ${\times10}^{-4}$ | 0.63 |
| 3 | BNNs-MPS-PEGDA | 1.05 ${\times10}^{-4}$ | 0.49 |
| 4 | LiBAMB-PEGDA-PETMP-MAPOSS/ PVDF-HFP | 2.8 ${\times10}^{-5}$ | 0.82 |
| 5 | Poly(B-GMA)-BDB | 4.7 ${\times10}^{-5}$ | 0.63 |
| 6 | ED-600-TA-bPEI | 2.7 ${\times10}^{-4}$ | 0.33 |
| 7 | IL-SE-B-HEMA | 3.8 ${\times10}^{-4}$ | 0.41 |
| 8 | PPLC copolymer | 1.1 ${\times10}^{-4}$ | 0.31 |

**Table S5**. Electrolyte model systems for MD simulations.

| # of molecule | GPE_TMAB | GPE_ETPTA | Solvent-free GPE_TMAB | Solvent-free GPE_ETPTA |
| --- | --- | --- | --- | --- |
| Li^+^ | 62 | 66 | 50 | 50 |
| TFSI^-^ | 62 | 66 | 50 | 50 |
| EMC | 307 | 330 | - | - |
| FEC | 436 | 469 | - | - |
| TMAB  matrix system | 1 | - | 1 | - |
| ETPTA  matrix system | - | 1 | - | 1 |
| Total # of atoms | 16225 | 17433 | 7068 | 7537 |

**Table S6.** Comparison of GPE_TMAB with the Reported Polymer Electrolytes in Terms of Cycle Number and Initial Discharge Capacity.^[12–19]^

| No. | Name | Cycle number | Initial discharge capacity [mAh/g] |
| --- | --- | --- | --- |
| 1 | GPE_TMAB | 800 | 140 |
| 2 | ePU@H | 500 | 124 |
| 3 | PACP-PEO@CF | 350 at 40 °C | 65 |
| 4 | CPEs-MNP | 300 at 60 °C | 151 |
| 5 | PPMI-3 SPE | 500 | 100 |
| 6 | PLNHFT | 500 | 135 |
| 7 | PEO-Ag | 500 | 140 |
| 8 | PEO-POF | 300 at 60 °C | 133.6 |
| 9 | DES-ETPTA | 700 | 122 |

**Supplementary Note S1.** MD simulation

The *NPT* ensemble was conducted for the initial equilibration of polymer TMAB matrix system, consisting of 40 TMAB, 245 MEA, and 77 IBA, and polymer ETPTA matrix system, consisting of 40 ETPTA, 264 MEA and 83 IBA, for 5 ns at 300 K, followed by 10 ns at 600 K. After constructing the initial TMAB and ETPTA matrix systems, the crosslinked structures were generated at a conversion ratio of 30% at 600 K. The *NVT* ensemble was employed for the cooling step of the crosslinked structures, with 10 ns at 600 K, followed by 5 ns at each temperature from 500 K to 400 K in 100 K intervals, and finally 25 ns at 300 K.

Upon completion of the crosslinking process, the model systems were constructed by combining the crosslinked polymer with the electrolyte components according to the compositions listed in Table S5. The *NPT* ensemble was conducted for 50 ns at 1 atm and 298.15 K. After *NPT* simulation, the *NVT* ensemble was performed for 50 ns at 298.15 K. Under an applied electric field of -0.1 V/Å along the *z*-direction, the *NPT* ensemble was further conducted for 5 ns at 1 atm and 298.15 K. Finally, the *NVT* ensemble was performed for 10 ns at 298.15 K. The radial distribution functions, coordination numbers, and mean squared displacements (MSD) were analyzed for the last 2.5 ns of *NVT* simulation under the applied electric field.

The self-diffusion coefficient was calculated from the following equation (S2):

$D= \frac{1}{6}\lim_{\Delta t\to\infty} \frac{\left\langle\left( \delta r \right)^{2} \right\rangle}{\Delta t}$ (S2)

where *δr* and Δ*t* are the changes in displacement and time, respectively, and the constant 6 is derived from three dimensions (*i.e.*, *x*, *y*, and *z*) and two [propagation directions](https://www.sciencedirect.com/topics/engineering/propagation-direction) (*i.e.*, forward and backward).

**Supplementary Note S2.** DFT calculation

An implicit environment was adopted by utilizing conductor-like screening model method with a dielectric constant of 22.45 for electrolyte condition (*i.e.*, 1 M LiTFSI in EMC/FEC (1:1 vol%)). The dielectric constant of electrolyte condition was calculated by mixing rule (dielectric constants = 2.4 (EMC), 79.7 (FEC)).^[20,21]^ The orbital cutoff was set to 3.7 Å (EMC, FEC, and crosslinked ETPTA), 4.0 Å (TFSI^-^), 4.1 Å (crosslinked TMAB) and 5.1 Å (LiTFSI and model systems for binding energies of Li^+^ or TSFI^-^ ). For the calculations of ESP charge and ESP isosurface of crosslinked polymer and the binding energies of TFSI^-^ anion with crosslinked polymer (*i.e.*, crosslinked TMAB and crosslinked ETPTA) and Li^+^ ion with TFSI^-^ or TFSI^-^-crosslinked polymer (*i.e.*, crosslinked TMAB and crosslinked ETPTA) and molecular orbital, generalized gradient approximation with Perdew-Burke-Ernzerhof functional was applied for the exchange-correlation energy.^[22]^ For the calculation of ESP charge of liquid electrolyte components (*i.e.*, Li^+^, TFSI^-^, FEC, and EMC), ESP isosurface of EMC and FEC, and binding energies of Li^+^ ion with solvation shell, Becke’s three-parameter hybrid exchange functional with Lee-Yang-Parr correlation functional was adopted for the exchange-correlation energy.^[23,24]^

The binding energies of Li^+^ ion with solvation shell were calculated from the following equation (S3),

${Li}^{+} binding energy= E_{\mathrm{total}}-\left( E_{solvation shell}+E_{\mathrm{Li}^{+}} \right)$ (S3)

where *E*_total_, *E*_solvation shell_, and *E*_Li+_ are the energy of the total system, different solvation shell (*i.e.*, 3EMC-1FEC and 2EMC-2FEC), and Li^+^ ion, respectively. In these calculations, all energy terms were obtained from single-point energy calculations. The binding energies of Li^+^ ion with TFSI^-^ anion or crosslinked polymer (*i.e.*, crosslinked TMAB and crosslinked ETPTA)-TFSI^-^ anion were calculated from the following equation (S4),

${Li}^{+} binding energy= E_{\mathrm{total}}-\left( E_{\mathrm{TFSI}^{-} or crosslinked polymer-\mathrm{TFSI}^{-}}+E_{\mathrm{Li}^{+}} \right)$ (S4)

where *E*_total_, *E*_TFSI- or crosslinked polymer-TFSI-_, and *E*_Li+_ are the energy of the total system, TFSI^-^ anion or different crosslinked polymer (*i.e.*, crosslinked TMAB and crosslinked ETPTA)-TFSI^-^ anion, and Li^+^ ion, respectively. The binding energies of TFSI^-^ anion with crosslinked polymer (*i.e.*, crosslinked TMAB and crosslinked ETPTA) were calculated from the following equation (S5),

${TFSI}^{-} binding energy= E_{\mathrm{total}}-\left( E_{crosslinked polymer}+E_{\mathrm{TFSI}^{-}} \right)$ (S5)

where *E*_total_, *E*_crosslinked polymer_, and *E*_TFSI-_ are the energy of the total system, different crosslinked polymer (*i.e.*, crosslinked TMAB and crosslinked ETPTA), and TFSI^-^ anion, respectively.

**Video S1.** Flame test of GPE_TMAB.

**Video S2.** Flame test of GPE_ETPTA.

**Video S3.** Flame test of LE.

**References**

[1]	P. Zhou, Y. Xiang, K. Liu, *Energy Environ. Sci.* **2024**, *17*, 8057.

[2]	M. Shanbedi, H. Shahali, A. A. Polycarpou, A. Amiri, *EES Batter.* **2025**, *1*, 385.

[3]	M. Kim, H. Moon, S. Kim, Y. Cho, T. Lee, S. Park, H. Kim, Y. H. Kwon, S. Hong, N. S. Choi, *Chem. Eng. J.* **2025**, *511*, 162079.

[4]	N. Zhong, C. Lei, R. Meng, J. Li, X. He, X. Liang, *Small* **2022**, *18*, 2200046.

[5]	Y. Ren, S. Chen, M. Odziomek, J. Guo, P. Xu, H. Xie, Z. Tian, M. Antonietti, T. Liu, *Angew. Chemie - Int. Ed.* **2025**, *64*, e202422169.

[6]	H. An, Q. Liu, J. An, S. Liang, X. Wang, Z. Xu, Y. Tong, H. Huo, N. Sun, Y. Wang, Y. Shi, J. Wang, *Energy Storage Mater.* **2021**, *43*, 358.

[7]	X. Zeng, P. Huang, J. Zhou, X. Wei, X. Wang, L. Chen, Y. Ding, J. Fu, *Chem. Eng. J.* **2024**, *487*, 150707.

[8]	B. Zhou, T. Deng, C. Yang, M. Wang, H. Yan, Z. Yang, Z. Wang, Z. Xue, *Adv. Funct. Mater.* **2023**, *33*, 2212005.

[9]	X. Wang, M. Wang, H. Chen, Y. Zhang, B. Niu, L. Tian, D. Long, *Chem. Eng. J.* **2024**, *496*, 153830.

[10]	Q. Zeng, Y. Liu, B. Wujieti, Z. Li, A. Chen, J. Guan, H. Wang, Y. Jiang, H. Zhou, W. Cui, S. Wang, L. Zhang, *Chem. Eng. J.* **2024**, *486*, 150298.

[11]	L. Zhao, Q. Dong, Y. Wang, G. Xue, X. Wang, Z. Li, H. Shao, H. Chen, Y. Shen, L. Chen, *Angew. Chemie - Int. Ed.* **2024**, *136*, e202412280.

[12]	S. Li, F. Pei, Y. Ding, X. Guo, X. Zhang, H. Tao, Z. He, H. Hu, L. Zhang, *Adv. Funct. Mater.* **2025**, *35*, 2415495.

[13]	M. Song, B. Xue, H. Wu, J. Li, W. Chen, W. Wang, W. Zhao, X. Gao, *Chem. Eng. J.* **2025**, *524*, 169043.

[14]	Z. Liu, K. Liu, K. Zhi, J. Luo, Z. Hu, Y. Zhang, *Chem. Eng. J.* **2025**, *504*, 158820.

[15]	D. Hu, H. Huang, C. Wang, Q. Hong, H. Wang, S. Tang, H. Zhang, J. Li, L. Hu, L. Jiang, X. Fu, J. Lei, Z. Liu, X. He, *Adv. Energy Mater.* **2025**, *15*, 2406176.

[16]	Q. Lv, Y. Jing, B. Wang, B. Wu, S. Wang, C. Li, L. Wang, L. Xiao, D. Wang, H. Liu, S. Dou, *Energy Storage Mater.* **2024**, *65*, 103122.

[17]	P. Zhai, N. Ahmad, S. Qu, L. Feng, W. Yang, *Adv. Funct. Mater.* **2024**, *34*, 2316561.

[18]	W. Liang, X. Zhou, B. Zhang, Z. Zhao, X. Song, K. Chen, L. Wang, Z. Ma, J. Liu, *Angew. Chemie - Int. Ed.* **2024**, *63*, e202320149.

[19]	C. Zhang, H. Zheng, L. Lin, J. Wen, S. Zhang, X. Hu, D. Zhou, B. Sa, L. Wang, J. Lin, Q. Xie, D. L. Peng, J. Lu, *Adv. Energy Mater.* **2024**, *14*, 2401324.

[20]	Q. K. Zhang, X. Q. Zhang, H. Yuan, J. Q. Huang, *Small Sci.* **2021**, *1*, 2100058.

[21]	A. Klamt, G. Schuurmann, *J. Chem. Soc., Perkin Trans.* **1993**, *2*, 799.

[22]	J. P. Perdew, K. Burke, M. Ernzerhof, *Phys. Rev. Lett.* **1996**, *77*, 3865.

[23]	A. D. Becke, *J. Chem. Phys.* **1993**, *98*, 5648.

[24]	P. J. Stephens, F. J. Devlin, C. F. Chabalowski, M. J. Frisch, *J. Phys. Chem.* **1994**, *98*, 11623.
